# Identification of integrin drug targets for 17 solid tumor types

**DOI:** 10.18632/oncotarget.25731

**Published:** 2018-07-10

**Authors:** Adith S. Arun, Clifford G. Tepper, Kit S. Lam

**Affiliations:** ^1^ Department of Biochemistry and Molecular Medicine, University of California Davis School of Medicine, UC Davis NCI-Designated Comprehensive Cancer Center, Sacramento, CA 95817, USA

**Keywords:** computational genomics, precision medicine, integrins, transcriptomics, therapeutic target selection

## Abstract

Integrins are contributors to remodeling of the extracellular matrix and cell migration. Integrins participate in the assembly of the actin cytoskeleton, regulate growth factor signaling pathways, cell proliferation, and control cell motility. In solid tumors, integrins are involved in promoting metastasis to distant sites, and angiogenesis. Integrins are a key target in cancer therapy and imaging. Integrin antagonists have proven successful in halting invasion and migration of tumors. Overexpressed integrins are prime anti-cancer drug targets. To streamline the development of specific integrin cancer therapeutics, we curated data to predict which integrin heterodimers are pausible therapeutic targets against 17 different solid tumors. Computational analysis of The Cancer Genome Atlas (TCGA) gene expression data revealed a set of integrin targets that are differentially expressed in tumors. Filtered by FPKM (Fragments Per Kilobase of transcript per Million mapped reads) expression level, overexpressed subunits were paired into heterodimeric protein targets. By comparing the RNA-seq differential expression results with immunohistochemistry (IHC) data, overexpressed integrin subunits were validated. Biologics and small molecule drug compounds against these identified overexpressed subunits and heterodimeric receptors are potential therapeutics against these cancers. In addition, high-affinity and high-specificity ligands against these integrins can serve as efficient vehicles for delivery of cancer drugs, nanotherapeutics, or imaging probes against cancer.

## INTRODUCTION

Integrins are heterodimeric receptors consisting of alpha and beta subunits. 18 α- and 9 β-subunits are transmembrane glycoproteins that combine non-covalently to form 24 distinct receptors. Integrins mediate cell adhesion to the extracellular matrix (ECM), and interact with distinct ECM proteins to remodel, migrate and respond to extracellular changes. Integrins function to regulate important biological processes such as proliferation, gene expression, cell survival and motility. The distribution of integrin proteins on the cell surface determines the type of ECM proteins able to bind to the cell and therefore impacts how a cell senses and responds to its microenvironment [1]. Different tissue types typically express a unique set of integrins on their cell surface. Integrin expression varies across cancer types and is associated with diverse extracellular milieu, migratory properties, and growth requirements of different tumors.

The myriad of cellular responses initiated by integrin signaling stem from their ability to differentially recognize distinct sets of ligands. In normal tissues, cell migration and morphogenesis are regulated by interactions of cell surface integrins with the extracellular matrix proteins, such as collagen, fibronectin, laminin, osteopontin, vitronectin, tenascin, fibrillin and VCAM-1 [1]. Tumors take advantage of integrin signaling in order to break adhesion-dependent movement and increase their invasive potential. Cancer cells exploit integrin-mediated processes to construct a microenvironment that promotes tumor invasion and metastasis. Integrins interact physically with various growth factors on the cell membrane [2, 3]. This pronounced interplay with growth factors and receptors are crucial towards regulating tumor progression and proliferation.

Ligated integrins enhance cell survival through several mechanisms, some of which include activation of the PI3K-AKT pathway, NF-κΒ signaling cascade, and p53 inactivation [4, 5]. Conversely, disrupting integrin-ligand interactions can induce apoptosis via integrin-mediated death (IMD), which is mediated by recruiting and activating caspase-8 [5]. Integrins expressed on tumor cells lead to tumor metastasis and progression, often by upregulating the activity of metalloproteinases (MMPs) and urokinase-type plasminogen activators [6, 7]. By interacting with the extracellular matrix of the secondary sites, tumor cells are able to establish residence and receive mitogenic input to continue their proliferation. Integrins are involved in regulating endothelial cell survival during angiogenesis and creating leaky vessels [8]. Therefore, antagonists against integrins facilitating these processes are essential to halting tumor metastasis and growth. Additionally, growth factors (e.g., fibroblast growth factor) concurrently bind both growth factor receptors (*e.g.*, fibroblast growth factor receptor) and integrins (e.g., integrin αvβ3), resulting in the promotion of tumor growth [2]. Antagonists against integrins will inhibit downstream signaling of growth factor dependent pathways due to the dynamic interplay between growth factor receptors and integrins [3].

Cancer stem cells (CSCs) are therapeutically resistant cells that reside within the main tumor population, and are responsible for long-term tumor survival and proliferation. Integrin signaling is crucial to maintaining stem cell properties within CSCs [1, 9]. Paired with growth factor receptors and through cooperation with tumor-promoting oncogene products (*e.g.*, WNT, MYC, AKT), integrins contribute to sustaining this aggressive subset of cells and regulating expression of stem cell specific markers. Drug resistance is dependent on tumor cells utilizing survival-inducing pathways, which can relate to ECM-integrin interactions. The integrin-ECM drug resistance strategy selects for cells that express integrins capable of adhering to the matrix proteins of the host organ thereby activating alternative pro-survival anti-apoptotic pathways.

Integrins are prime targets for imaging and therapy in cancer [10]. We have previously reported the use of a one-bead one-compound (OBOC) combinatorial library method to discover ligands against integrins (α4β1, α3β1, and αvβ3) that are overexpressed in various cancers, and use them as vehicles to delivery of theranostic agents or nanoformulated drugs to tumors [11–14].

Small-molecule drugs and biologics directed against overexpressed cell surface proteins such as EGF receptor and HER2 in tumors have been effective in treating cancers [15]. In the advancing field of immunotherapy, programmed cell death ligand-1 (PD-L1) inhibiting drugs are more successful in the subset of patients with their tumors overexpressing PD-L1 [16–18]. Similarly, the same principle should be applied to integrin-based therapies. Identification of integrins overexpressed in various tumor types will enable us to focus our drug development effort on these targets. To achieve this, curating data from large clinical databases will be much more relevant than studying integrin expression in a limited number of established cancer cell lines.

In this study, we sought to identify actionable integrins in 17 different cancer types by computational analyses of the transcriptome data obtained from The Cancer Genome Atlas (TCGA). Using RNA-Sequencing (RNA-Seq) expression data, overexpressed integrins were found, followed by a prioritization scheme involving the application of a ranking metric and filtering by FPKM absolute expression level. In addition, immunohistochemistry data from the Protein Atlas was integrated for validation of results and the most prominent integrin heterodimers on each cancer were predicted.

## RESULTS

### Profiles of integrin gene expression in 17 different cancers

**Figure 1:**
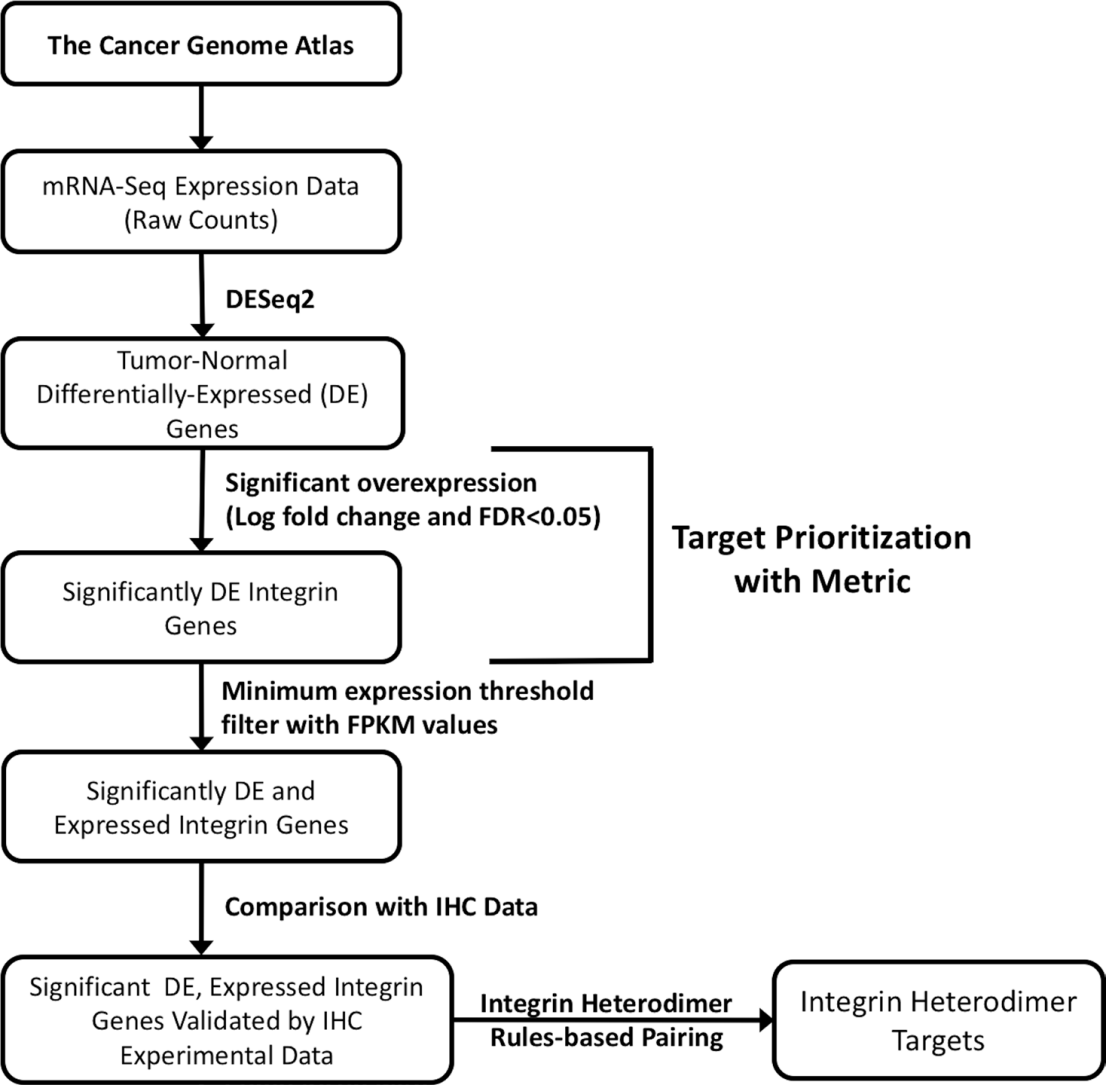
Schematic flowchart depicting the strategy for selecting integrin drug targets Transcriptome profiling data for 17 cancer types from the TCGA was used for analysis. RNA-seq data (raw counts) were retrieved from the Genomic Data Common using TCGAbiolinks. Target prioritization was then accomplished by applying Metric to define integrin subunit genes significantly overexpressed in tumor samples (logarithmic fold change, FDR < 0.05). Subsequently, viable, individual subunit drug targets were selected by filtering the results for integrin transcripts passing a minimum threshold of expression (FPKM values) and by comparison with immunohistochemistry data (IHC) for protein-level expression expression of the corresponding subunits. Through rules-based pairing of subunits, possible protein integrin drug targets are proposed. See *Materials and Methods* for more detailed descriptions of each step.

The strategy that was employed to determine integrin anti-cancer drug targets across 17 different tumor subtypes is depicted in the flowchart in Figure 1. RNA-seq expression data for all integrin genes was sourced from The Cancer Genome Atlas (TCGA), and differential expression analysis of expressed integrin genes was performed with DESeq2. The differentially expressed (DE) genes were then prioritized in a ranking scheme. Two unique criteria were incorporated into a mathematical Metric, namely, the *1)* logarithmic fold change of expression between cancer and normal samples, and *2)* false discovery rate. The following sections detail the results of the selection process and the outcome of the individual targetable integrin subunits as well as the obligated integrin heterodimers. Accompanying this ranking system, the expressed integrin genes were filtered by the total level of expression, denoted by FPKM values, also obtained from TCGA. By comparing the predicted integrin targets against existing experimental immunohistochemistry data obtained from The Protein Atlas, the resultant targets were confirmed. Then, using the rules of heterodimeric integrin subunit pairing, therapeutic target integrins for each cancer were predicted.

**Figure 2:**
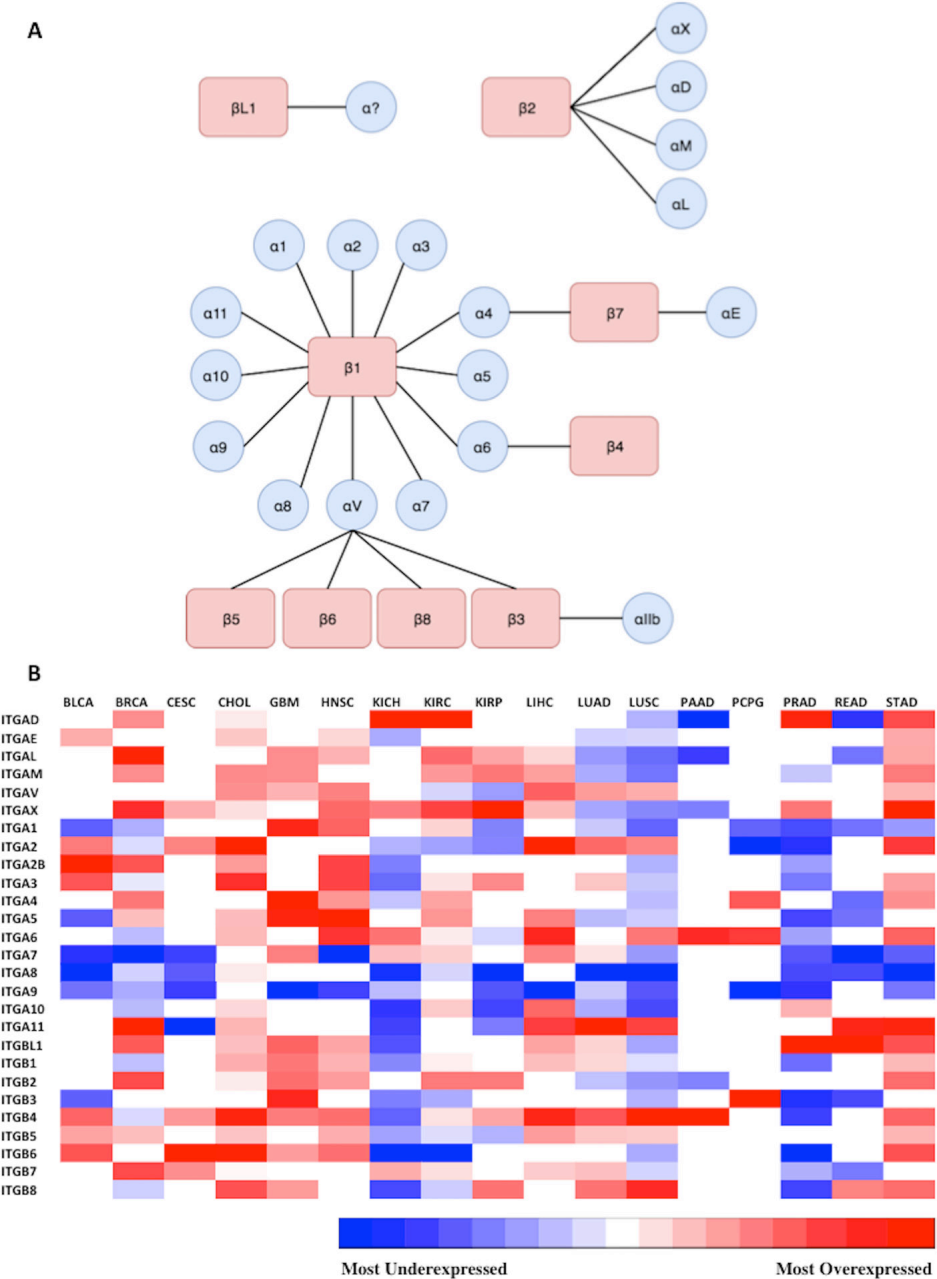
Representation of expression of 27 integrin subunits across 17 cancer types (**A**) Possible combinations of integrin subunits to form 24 biologically functional integrin heterodimers. The different possible combinations of alpha and beta subunits capable of forming heterodimeric integrin proteins are displayed. 24 unique heterodimeric receptors can be formed from 9 beta subunits and 18 alpha subunits. The integrin beta-like 1 subunit has been characterized, but thus far an alpha subunit binding partner has not been identified, and is represented by a question mark. (**B**) Diagrammatic visualization of all integrin subunits across surveyed cancer types. Differential expression of integrin subunit genes was determined by comparing the expression of a subunit in tumor samples versus normal samples as outlined in *Materials and Methods*. The gradient from blue to red represents the magnitude of differential expression of tumor versus normal; darkest blue and the darkest red indicates the most underexpressed and overexpressed gene in each cancer type, respectively. The relative log fold change expression of each integrin subunit within each cancer type is depicted in the heatmap matrix above. White boxes represent void values due to the false discovery rate being greater than 0.05. RNA-Seq data (transcript counts) was obtained from TCGA and differential expression analysis was performed in R (as detailed in *Materials and Methods*). The cancer types examined and their abbreviations (Supplementary Table 1) are as follows (https://gdc.cancer.gov/resources-tcga-users/tcga-code-tables/tcga-study-abbreviations): Urothelial Bladder Carcinoma (BLCA), Breast Invasive Carcinoma (BRCA), Cervical Squamous Cell Carcinoma and Endocervical Adenocarcinoma (CESC), Glioblastoma Multiforme (GBM), Head and Neck Squamous Cell Carcinoma (HNSC), Kidney Chromophobe (KICH), Kidney Renal Cell Carcinoma (KIRC), Kidney Renal Papillary Cell Carcinoma (KIRP), Liver Hepatocellular Carcinoma (LIHC), Lung Adenocarcinoma (LUAD), Lung Squamous Cell Carcinoma (LUSC), Pancreatic Adenocarcinoma (PAAD), Paraganglioma and Pheochromocytoma (PCPG), Prostate Adenocarcinoma (PRAD), Rectum Adenocarcinoma (READ), Stomach Adenocarcinoma (STAD).

The overall goal of this study was to define potentially targetable integrins present in each of 17 types of solid cancer, using clinical data sets obtained from a public database. Our approach to selecting and prioritizing actionable integrins was based on satisfying the following criteria: *1)* overexpressed in cancer (relative to normal tissue), *2)* high ranking Metric score, and *3)* having at least a moderate level of absolute expression. As described in the Introduction, the complete repertoire of integrin receptors is constituted of 27 integrin genes encoding for 18 α- and 9 β-subunits. These α- and β-subunits form 24 different known obligated heterodimers of integrins (Figure 2A). RNA-Seq transcriptome data (transcript counts) was sourced from the respective TCGA studies and computational analyses performed to identify integrin genes that were overexpressed in tumor samples and then to prioritize integrins and predict the heterodimers that would most likely be present in each cancer. Accordingly, as the first step, the expression of the 27 integrin genes in each of the studies’ datasets were analyzed to define integrin transcripts that were differentially expressed in each cancer (*i.e.*, relative to normal tissue controls, FDR < 0.05) (Supplementary Table 1). Integrin genes that did not exhibit statistically-significant differential expression (*i.e.*, FDR > 0.05) are indicated by blank, or white boxes (Figure 2B, Supplementary Table 1). The results were consolidated and presented as a heatmap matrix to depict the relative level of differential expression (*i.e.*, fold changes) (Figure 2B).

As shown, the profile and magnitudes of integrin differential expression varies considerably across the different cancer types. However, similarities in integrin expression across the cancer types are evident. Tumors reveal several distinct patterns in alteration of integrin expression. For instance, *ITGA9* is the most widely underexpressed subunit across the 17 profiled cancers, ranging from −1.41 to −5.89 fold reduction in cancer compared to normal. In contrast, *ITGAX* is the most widely overexpressed subunit among the profiled cancers (1.54 to 8.11 linear fold change). Aggressive carcinomas with low 5-year survival rates display unique and diverse expression patterns. For example, cholangiocarcinoma (CHOL) overexpresses a large variety of subunits (*e.g. ITGAV, ITGA1, ITGA4, ITGA5, ITGA11, ITGB1, ITGB2, ITGB6*), whereas, the equally deadly pancreatic adenocarcinoma (PAAD) overexpresses a very limited set of integrins, including *ITGA6 and ITGB4*. Although both CHOL and PAAD have low 5-year survival rates, they display completely different integrin expression profiles. Interestingly, tumors originating from the same organ such as kidney chromophobe (KICH), kidney renal cell carcinoma (KIRC) and kidney papillary renal cell carcinoma (KIRP) share few integrin expression features with one another (Figure 2B).

**Figure 3:**
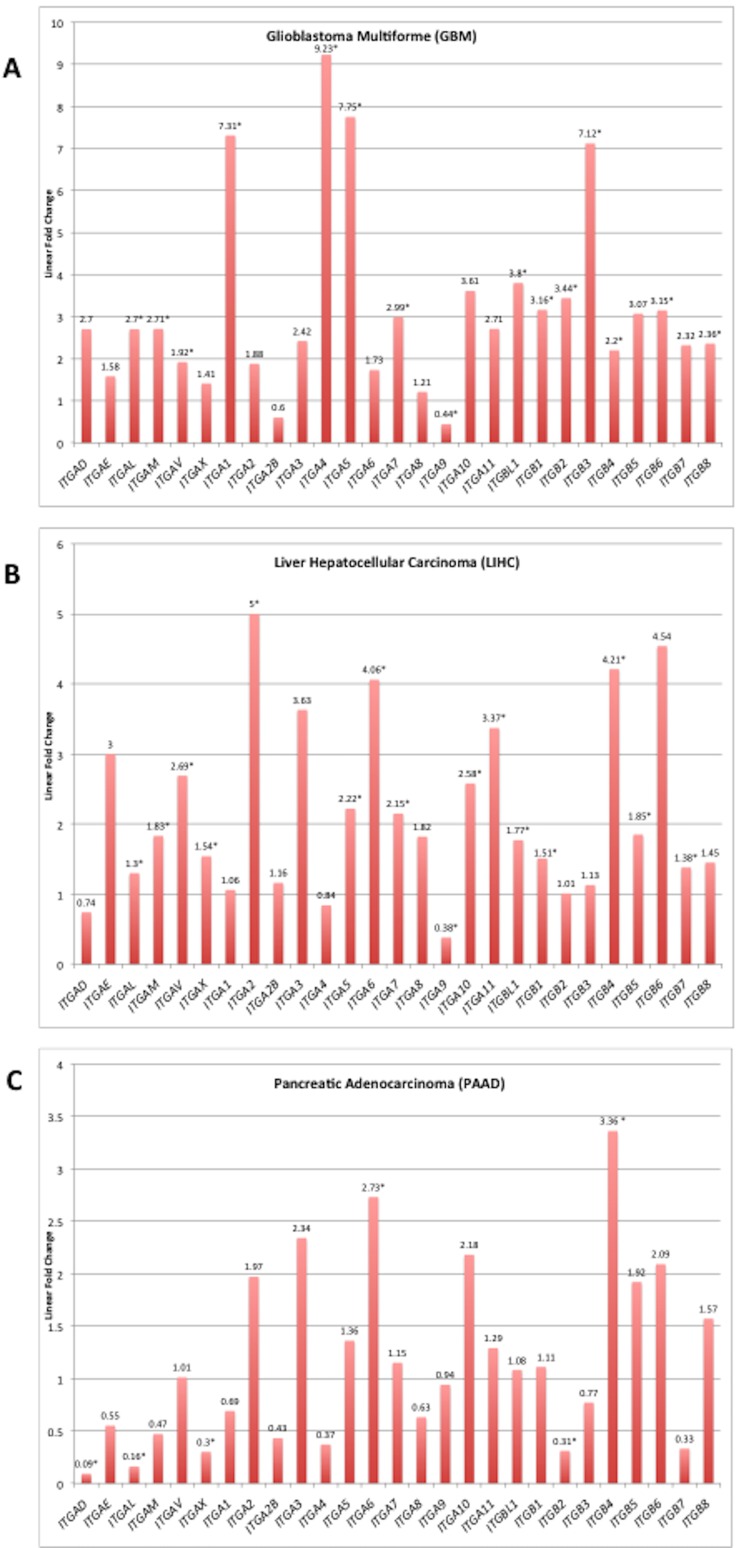
Integrin subunit expression for selected tumor types Analysis for differentially expressed genes (tumor vs. normal) was performed on RNA-Seq expression data obtained for each TCGA cancer site project. Linear fold changes for each integrin in GBM, LIHC, and PAAD are presented in the bar graph. The y-axis is the linear fold change in expression, and each bar represents a different integrin subunit. The linear fold change threshold represented by the line y = 1 indicates the threshold for differential expression in either direction; values below this line are underexpressed in cancer and values above are overexpressed. Asterisks following the annotated value represent statistically significant alterations (FDR < 0.05). Source material for these graphs is Supplementary Figure 1. The complete FDR, *p*-value, and logarithmic fold change values can be found in Supplementary Table 2.

As examples, the linear fold change in expression levels (tumor vs. normal) for the integrin subunits are displayed for glioblastoma multiforme, hepatocellular carcinoma, and pancreatic adenocarcinoma (Figure 3). The range of expression ratio (tumor vs normal) varied across the three depicted cancer types, and was determined to be 8.79, 4.26, and 3.27 for glioblastoma multiforme, hepatocellular carcinoma, and pancreatic adenocarcinoma, respectively. Interestingly, 25 of the 27 surveyed integrin subunit genes, i.e. 93% of the total surveyed integrin genes, exhibited elevated levels of expression in glioblastoma multiforme compared to normal brain tissue. However, only 52% of the 27 surveyed genes reached statistical significance (FDR < 0.05). Pancreatic adenocarcinoma, on the other hand, was found to overexpress 51% of the surveyed integrin subunits yet only 7.4% (2 of the 27 genes) reached statistical significance.

### Ranking overexpressed genes using a scoring system to determine best potential targets

**Figure 4:**
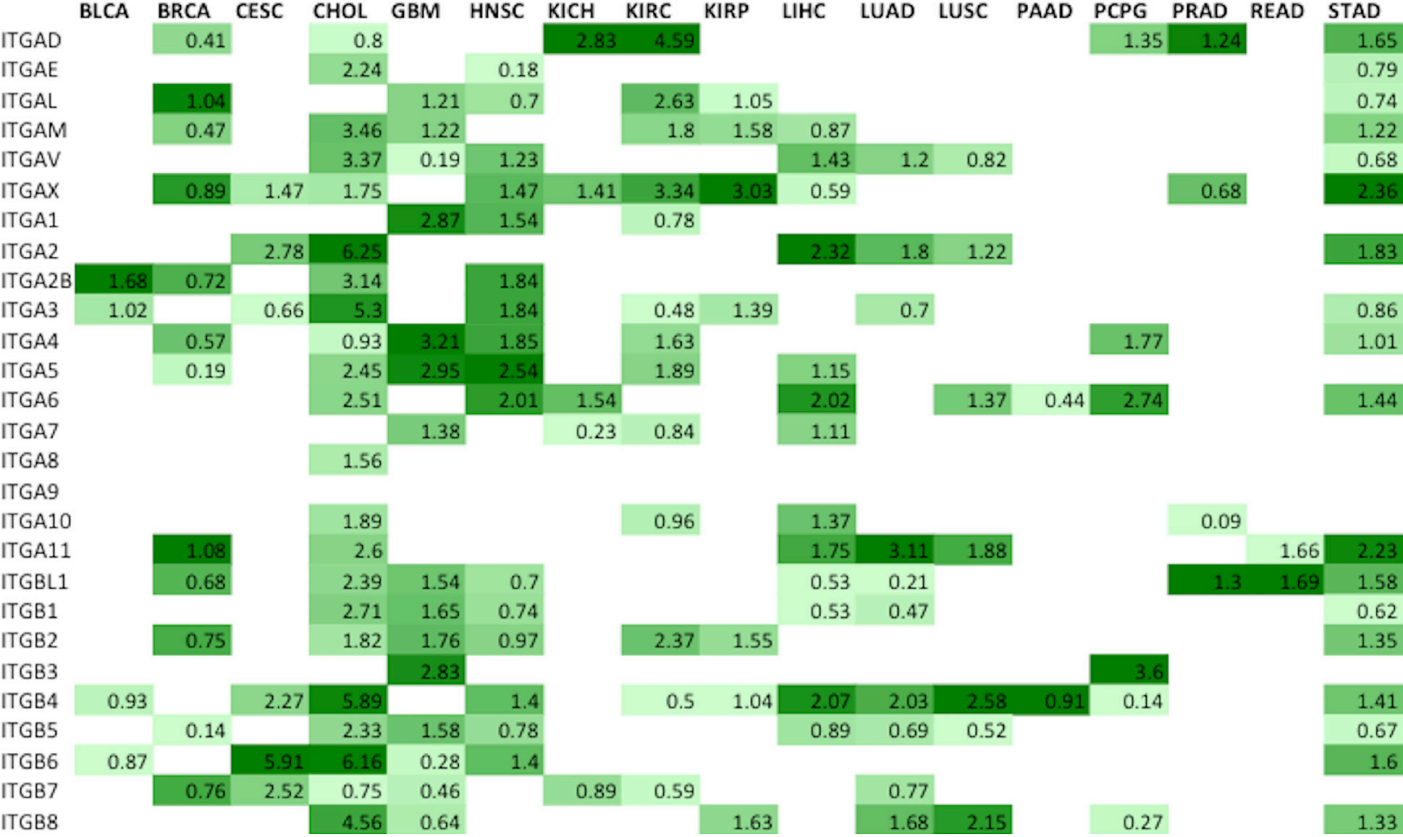
Metric ranking of the best potential therapeutic integrin targets A scoring system was developed and applied to the RNA-Seq data in order to predict the best integrin drug targets specific to each cancer type (*Materials and Methods*). The two components that comprised Metric were the logarithmic fold change value and the false discovery rate (FDR). In order to generate the Metric values, both components were considered for each subunit for each tumor type and applied to a formula described in *Materials and Methods*. Colored on a spectrum from light to dark green, the lowest values are colored lighter shades while the higher values are colored darker shades. Higher values indicate more promising drug targets based on the following criteria: high level of differential expression (logarithmic fold change), acceptable FDR values (*p* < 0.05).

A ranking system for determining the best potential integrin drug targets was developed (Figure 4); the following elements were considered: statistically significant differential expression (FDR < 0.05), and magnitude logarithmic fold change. For ranking overexpressed genes, the scoring system used consisted of a weighted average of logarithmic fold change for that gene in that specific cancer. The resulting ranks are reported on a scale with a lower boundary of zero and no forced upper limit. Higher values correspond to the more optimal targets (Figure 4). At least two subunits – one α, one β – in each surveyed cancer were identified as potential drug targets. Although liver hepatocellular carcinoma (LIHC) had 24 overexpressed genes, only 15 were ranked when considering the false discovery rates (Figure 3). Promising targets, represented by colored boxes, had high logarithmic change values, with FDR < 0.05 (Figure 4). This ranking system is a mathematical screen and the biological basis for selecting targets will be introduced in the following sections and includes FPKM levels, subunit pairing rules, and immunohistochemistry results.

### Absolute expression level of ranked integrin genes

**Figure 5:**
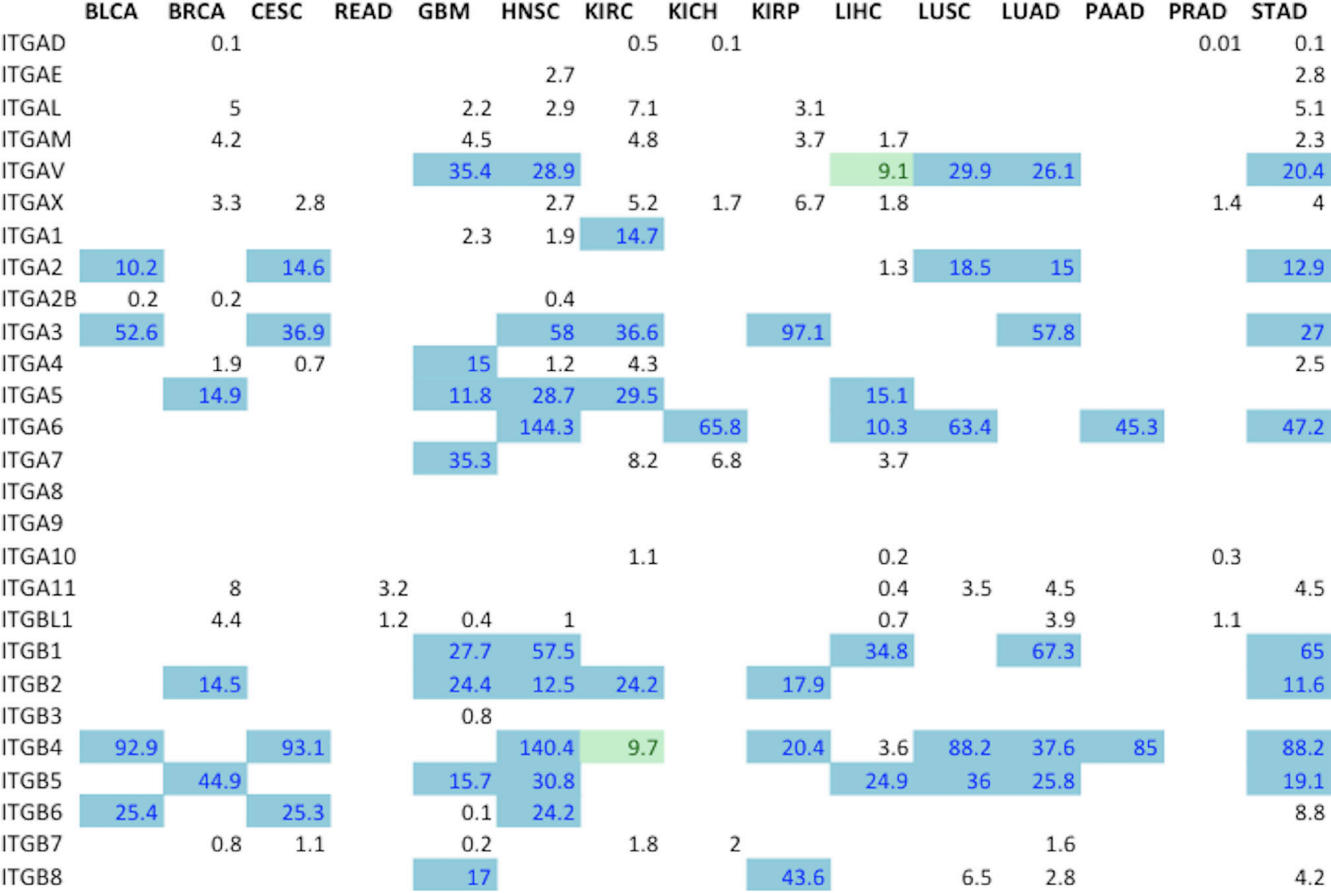
Expression levels for all ranked integrin subunit genes FPKM expression values for all the ranked integrin subunits (Figure 2B) were obtained from TCGA Expression Graphs hosted at The Protein Atlas (https://www.proteinatlas.org/). FPKM values ≥ 10 (Blue) denote that the level of expression of the gene is significant enough to represent a targetable receptor. The boxes colored in green indicate FPKM values very close to the cut-off, and are also considered targetable subunits.

In addition to considering integrin gene differential expression as a major selection criterion, candidate integrin subunits were further filtered on the basis of transcript expression levels. Normalized gene-expression level reported in FPKM (*F*ragments *P*er *K*ilobase of transcript per *M*illion fragments mapped) values [19] for all ranked integrin gene subunits were found in the published data housed within the Protein Atlas (Figure 5). Integrin subunits having average FPKM values ≥ 10 were considered to be actionable by virtue of being expressed at an easily detectable level of expression. In the process of determining the heterodimeric proteins that serve as prime drug targets, the ranked subunits according to the metric (Figure 4) were filtered using FPKM values (Figure 5). The absolute integrin expression level, ≥10 FPKM cut-off for moderate expression, determines whether the cell expresses a significant amount of the integrin gene of interest such that its inhibition would disrupt cellular function. Without there being a significant level of absolute expression in tumor cells, differential expression analysis alone could yield a sub-optimal target.

### Pan-cancer immunohistochemical analysis of integrin subunit overexpression

**Figure 6:**
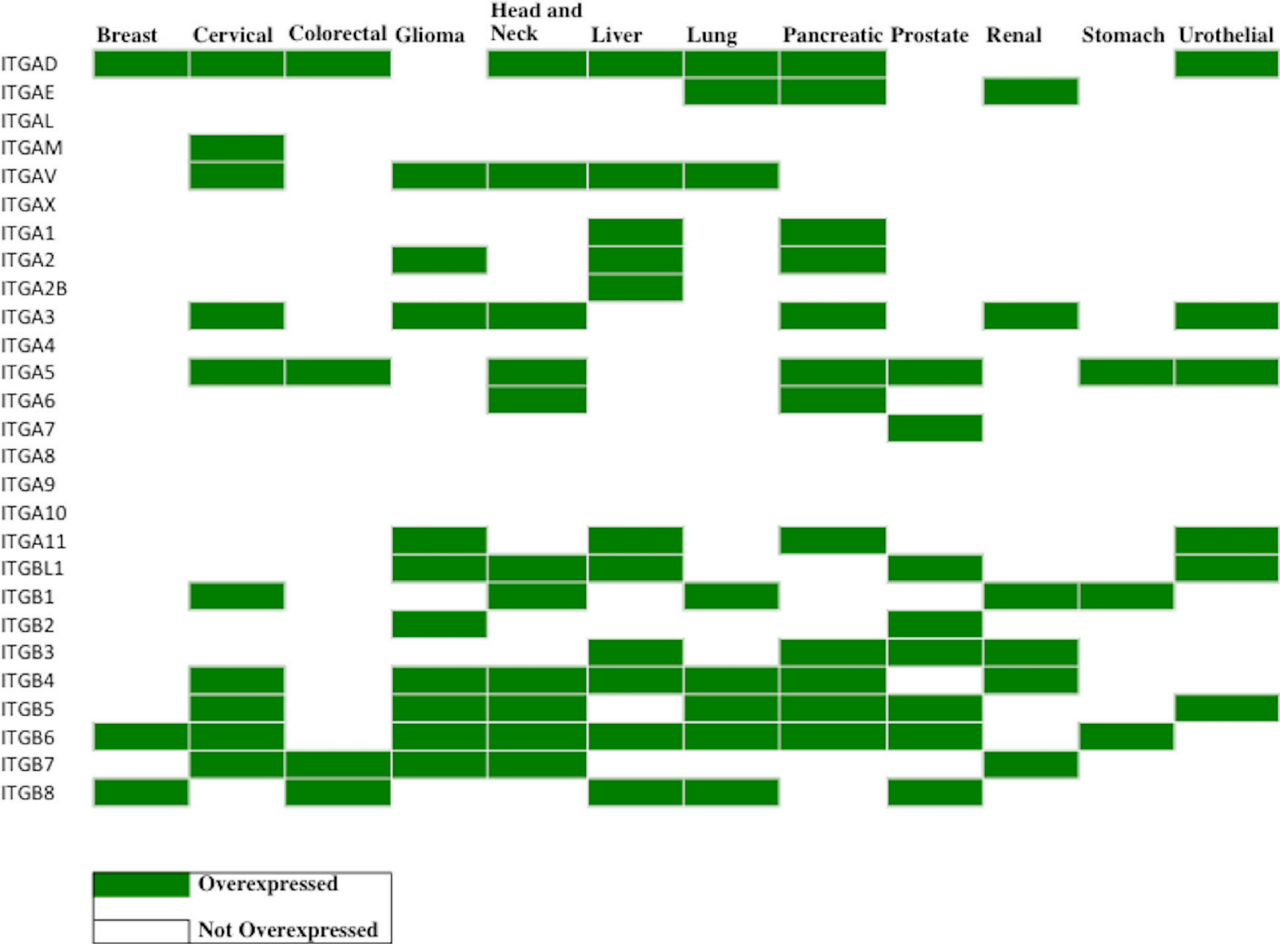
Profiling of integrin protein overexpression across 12 cancer types Immunohistochemistry (IHC) data from The Human Protein Atlas (https://www.proteinatlas.org/humanpathology) was analyzed for integrin subunit protein expression in each cancer type (*Materials and Methods)*. The values of the translated quantitative IHC expression levels is shown in Supplementary Table 3A in the format (tumor, normal). Overexpression was determined when the tumor value exceeds the normal value. In the table above, the cancers exhibiting overexpression of the indicated integrin subunits are displayed in green. A blank box indicates that either there was no overexpression of the integrin compared to normal tissue samples or that the Protein Atlas did not perform IHC analysis of that integrin subunit (IHC analysis was not performed for *ITGA4* and *ITGA10*). Since the cancer types are broadly categorized by organ site in the Protein Atlas, data are not available for certain cancer sub-types, specifically renal (KIRC, KICH, KIRP), lung (LUAD, LUSC), colorectal (COAD, READ), PCPG and CHOL. Immunohistochemistry data was collected from the Protein Atlas, and only the data from validated antibodies were used for analysis. Supplementary Table 3B displays the antibodies used and sample sizes. A number of integrin subunits only displayed data from one antibody, thus it was used as default regardless of validity (*ITGAD, ITGAL, ITGA1, ITGA7, ITGA8, ITGA9, ITGA11, ITGBL1, ITGB1, ITGB3, ITGB7* have data reported by one antibody only). Additionally, data for *ITGA4* and *ITGA10* subunits are intentionally left empty because the Protein Atlas did not report any results for these subunits.

As one approach to validating that the expression of the candidate integrins at mRNA level were also expressed at the protein level, and elevated, we analyzed immunohistochemical data from the Human Protein Atlas (https://www.proteinatlas.org/humanpathology). The overexpressed integrins can be highlighted by integrating the results of RNA-seq differential expression (Figure 4) and gene expression level filtering (i.e., FPKM) (Figure 5) with IHC data (Figure 6). In the IHC database, groups of cancers are not sub-classified, but rather grouped together by the organ from which they originate. Thus, comparisons of expression between sub-types of cancers originating from the same organ (*eg.*, Renal denotes KIRC, KIRP, and KICH) is not possible. IHC does not discriminate between different cancer subtypes within the same organ when pathologically examining samples for integrin expression. Extensive similarities between the IHC and RNA-seq expression data, however, do exist. In both methods of analysis, *ITGA6* is overexpressed in head and neck, lung, liver, and pancreatic cancers.

Cholangiocarcinoma is often binned with liver cancer; however, IHC data from the Protein Atlas does not distinguish or recognize this difference. *ITGAV*, in both IHC and RNA-seq, is overexpressed in liver cancers, gliomas, head and neck cancers, and lung cancers. Validated with both IHC and RNA-seq, *ITGB6* is overexpressed in cervical cancer, liver cancer/cholangiocarcinoma, head and neck cancer, and stomach cancer. However, differences between the IHC and RNA-seq data do exist. For example, in the case of *ITGB2*, IHC indicates that *ITGB2* is only overexpressed in gliomas and prostate cancers whereas RNA-seq identifies *ITGB2* elevation in breast cancer, glioma, head and neck cancer, renal cancer and stomach cancer.

### Identification of cancer type-specific therapeutically actionable heterodimeric integrin receptors

**Figure 7:**
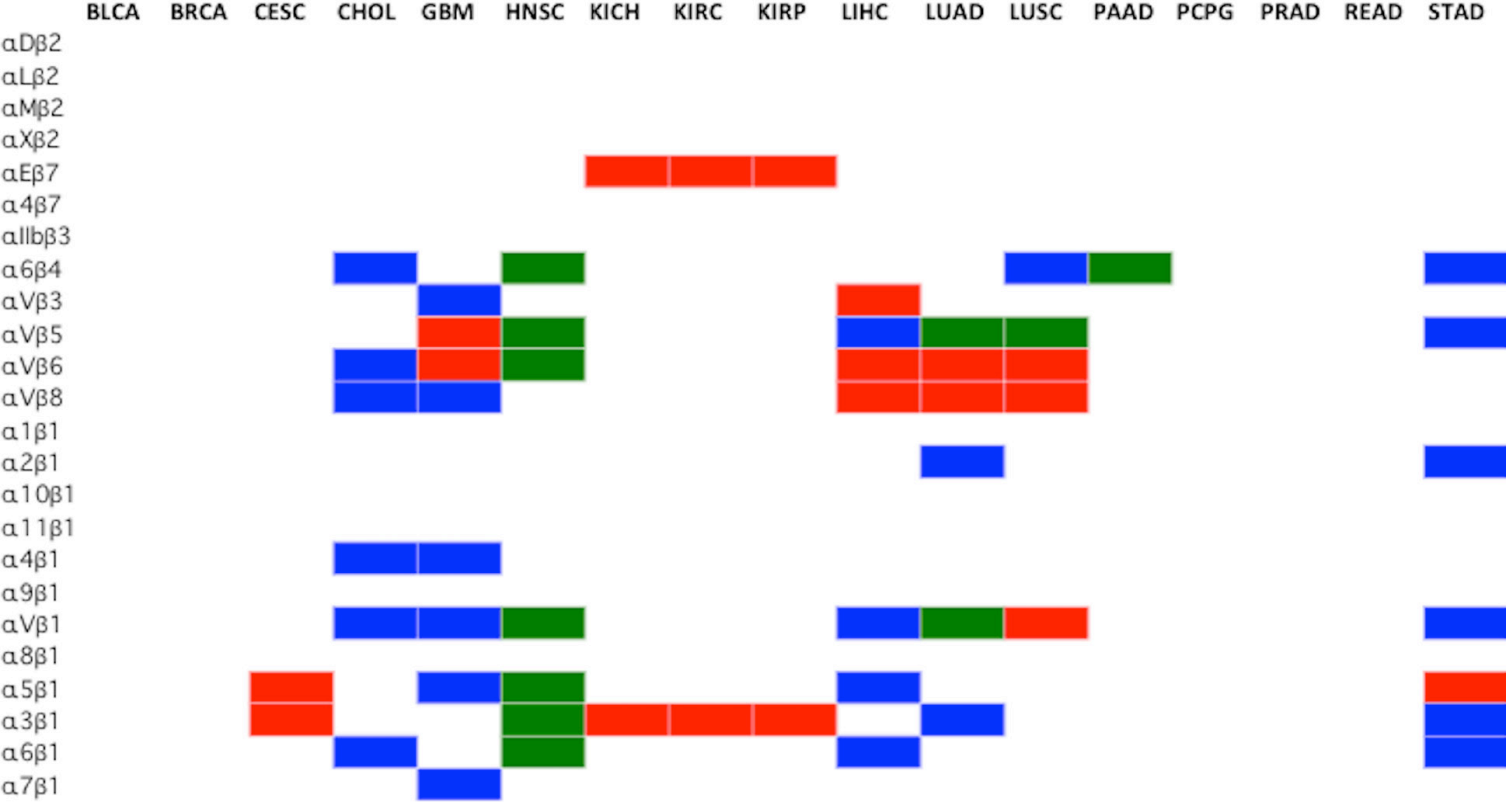
Selection of cancer type-specific therapeutically actionable integrin heterodimer receptors The therapeutically actionable receptors are based on the highest ranked, according to Metric, and FPKM-filtered subunit genes. The subunits that passed the Metric and FPKM filters were combined, according to the integrin pairing rules (Figure 2A), to form any of the 24 possible obligated heterodimeric integrins displayed on the cell surface. Blue indicates integrin heterodimers identified as targets through differential expression analysis of TCGA data only, Red indicates results obtained from immunohistochemistry only (Figure 5), and Green indicates that both methods agree. The cancer types (BLCA, BRCA, CESC, KICH, KIRP, KIRC, PCPG, PRAD, READ), did not have viable heterodimer integrin options based on the FPKM filtered ranked genes from RNA-seq analysis. Renal cancer data from IHC was broadly applied to KICH, KIRP and KIRC. Similarily, immunohistochemistry lung cancer data was applied to LUSC and LUAD.

In previous discussion, individual integrin subunits were identified as potential targets (Figure 4); however, the actual druggable integrins are the heterodimeric receptors. Absolute expression level selection criteria resulted in only the subunits highlighted in blue in Figure 5 being considered for pairing of integrin subunits. These gene subunits were combined using the existing rules of obligated integrin pairing (Figure 2A) to determine heterodimeric receptor drug targets for each cancer (Figure 7).

Using the aforementioned selection parameters outlined in Figure 1, 10 of the 17 profiled cancers have no viable heterodimer options, according to the computational analysis seen in blue (Figure 7), as the rules for pairing did not allow for any of the FPKM expression-filtered gene subunits to interact. Only subunits that were ranked highly according to the Metric and higher than 10 FPKM were considered viable partners for heterodimer pairing. Imposing the rules of integrin pairing (Figure 2A) further constrained the possible combinations of therapeutically actionable targets identified by the computational analysis.

The integrin protein drug targets identified through the computational method introduced in this study were compared against the integrin targets identified by immunohistochemistry staining (Figure 6). Since immunohistochemistry (IHC) data only stained for individual subunits, the rules for subunit pairing were observed in determining the integrin protein targets. The IHC data, colored red, identified many targets for lung, cervical and liver cancer that were not supported by the computational analysis of RNA-seq data. Conversely, the computational analysis of the RNA-seq data identified many targets for brain, stomach cancer and cholangiocarcinoma that were not supported by IHC data. It is important to note that IHC tissue analysis for *ITGA10* and *ITGA4* expression level are not yet available at this moment in the literature and therefore results for these integrins are not possible to report at this time. Additionally, IHC data is reported by organ, and thus the data from lung and kidney cancers were broadly applied to the subtypes profiled by TCGA (LUAD, LUSC, KIRP, KIRC, KICH). Both the IHC and the computational analysis methods (Figure 6, shown in green) however, do agree on all the identified head and neck squamous cell carcinoma and pancreatic adenocarcinoma integrin targets.

**Figure 8:**
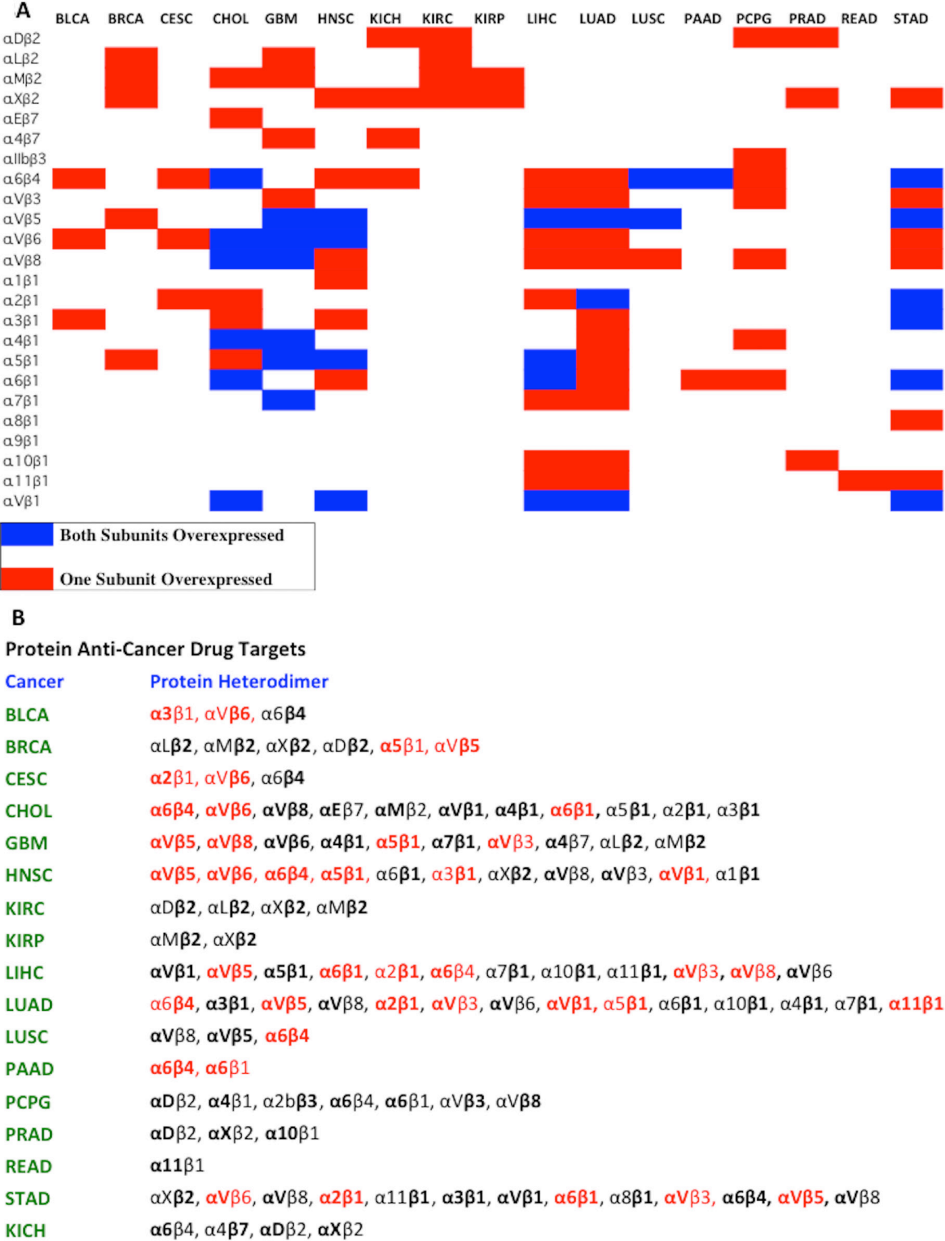
Pausible integrin heterodimers as cancer therapeutic targets (**A**) A chart representing the plausible integrin heterodimers is shown. The red boxes indicate that only one subunit was overexpressed and the blue boxes indicates that both subunits were overexpressed. (**B**) The full expanded version of Part A is depicted in Part B. A comprehensive map of heterodimeric integrin targets for 17 different tumor types based on the computational analysis is presented. The bolded subunits represent subunits that are overexpressed compared to normal samples (Figure 4), and are absolutely expressed at a level greater than 10 FPKM (Figure 5). The integrin heterodimers that are colored in red represent receptors for which previously published data exists regarding differential expression and/or usefulness in cancer treatment and diagnosis.

The actionable integrin receptors charted in Figure 7 represent the set of possible heterodimers where both paired subunits are significantly overexpressed in tumor samples compared to normal and have FPKM value greater than 10. Overexpression of both subunits in a heterodimeric pair may not be needed for a viable drug target. It is conceivable that high expression level of mRNA encoding an integrin subunit could still drive the entire heterodimer to a high level of expression, even if the partner chain is at normal level. Using this relaxed criteria, many additional plausible therapeutic integrin heterodimers emerge. The full-range of possible therapeutically actionable heterodimeric integrin receptors stemming from the computational analysis are charted in Figure 8.

Red colored integrin heterodimers in Figure 8B indicates that there exists prior studies that identify the receptor as a valid target. Bolded text (Figure 8B) represents subunits with high Metric ranking and FPKM > 10. Subunits in normal font indicates standard expression, high expression but without a proper FDR value, or high expression but FPKM < 10. For easy visualization, Figure 8A serves as a graphical representation of the plausible heterodimeric integrin targets. Integrin α3β1 is expressed in high levels in bladder cancer [20]. It has been previously shown that targeting αv integrins reduces malignancy in bladder cancer [21]. Integrin α5β1 and integrin αvβ5 are shown to be viable targets in invasive breast carcinoma [22]. Cervical squamous cell carcinoma and endocervical adenocarcinoma overexpress integrins α2β1 and αvβ6 [23, 24]. Cholangiocarcinoma overexpresses β4 integrin subunit [25]. A migratory and highly invasive cholangioacarcinoma phenotype is closely linked to expression of the integrin α6 subunit, where higher expression is associated with more invasive properties [26]. By the rules of integrin pairing (Figure 2A), the β4 subunit must pair with α6 subunit, thereby supporting the data displayed in Figure 8B. Additionally, integrin αvβ6 has been shown to be overexpressed in cholangiocarcinomas [27]. The αv integrins are prime targets for glioblastoma therapeutic strategies and are highly overexpressed in malignant samples compared to normal samples: integrins αvβ5, αvβ8, and αvβ3 [28–30]. In addition, α5β1 has been previously shown to be key in regulating growth and tumor progression in glioblastoma [31]. Head and neck squamous cell carcinoma has been extensively studied and integrins αvβ5, αvβ8, α5β1, α6β4, αvβ8, and αvβ6 have clearly been shown to be overexpressed receptors [32, 33]. Further evidence supports that for head and neck squamous cell carcinomas, the β1 integrins in general are necessary for maintaining proper tumor processes and growth [34, 35]. Liver hepatocellular carcinoma overexpresses many integrin receptors including the previously established integrins αvβ3, αvβ5, α2β1, α5β1, α6β1, and α6β4 [36–40]. β1 and β8 subunits overexpression, in general, have been implicated in facilitating liver hepatocellular carcinoma metastases and progression [40, 41]. Lung adenocarcinoma integrin drug targets that have been previously vetted are integrins αvβ3, αvβ5, α2β1, αvβ1, and α6β4 [42–44]. Additionally, integrin α11β1 has been shown to enhance tumorigenicity and metastasis in lung adenocarcinoma [45]. Integrin α5β1 is essential for lung tumor progression as it recognizes fibronectin and is a potential anti-lung cancer therapeutic candidate [46]. Previous research has identified integrin α6β4 to be crucial in squamous cell lung carcinoma development and growth [47]. Similarly, integrin α6β4 is overexpressed and a prime therapeutic target in pancreatic adenocarcinomas [48, 49]. It has also been previously shown that integrin α6β1 overexpression increases metastatic capability of pancreatic cancers [50]. The overexpression of αv integrins (αvβ3, αvβ5, αvβ6) serves as key prognostic, and therapeutic targets in stomach cancers [51, 52]. Overexpression of integrins subunits α2 and α6 increase the invasive potential of gastric cancers and prime the tumor cells for invasion into surrounding tissues [53].

## DISCUSSION

Through computational analysis of RNA-seq data and comparison to immunohistochemistry data, overexpressed integrins in 17 solid tumors were characterized. Individual integrin subunits identified in this study may serve as potential drug targets for the development of cancer therapeutics. Inhibition of individual subunits is expected to disrupt cellular signaling and acts to halt tumor metastatic processes, angiogenesis and growth. The heterodimeric integrin drug targets identified in this study are overexpressed in their respective tumor types and therefore are prime targets for therapeutic development. Integrins are evolutionarily conserved, and also conserved by a tumor indicating the importance of integrins for sensing and interacting with the external environment. Through our bioinformatics and mathematical analysis scheme, a new method for selecting integrin therapeutic targets has been introduced. This study contributes a pattern-oriented method for pinpointing specific integrin targets, which can streamline the drug discovery process. Integrin heterodimers, with high expression on tumor cells compared to normal cells, outlined in this study can also serve as cell surface targets for nanodelivery of drugs and imaging agents.

The results of our analyses demonstrate that each type of cancer examined possesses a distinctive integrin gene overexpression signature. While this is not unexpected, the results serve as an additional confirmation of previously targeted integrins and highlight those that should potentially be prioritized. The results are based on transcriptome data, which has its limitations. Most notably, it is not completely indicative of protein expression. However, these findings emphasize the need for therapeutic approaches that are individualized for cancer type, and for the patient, if appropriate clinical molecular testing is available. For example, cholangiocarcinoma (CHOL) overexpresses a large variety of subunits possibly to increase migration and promote metastasis whereas pancreatic adenocarcinoma (PAAD) overexpresses a very limited set of integrins (Figure 2B). The relatively few expression features shared by KICH, KIRC, and KIRP indicate that there may be major differences in tumor biology in kidney cancers. (Figure 2B). Although the two most common types of non-small cell lung cancer, lung adenocarcinoma (LUAD) and lung squamous cell carcinoma (LUSC), originate from distinctive cell types, they have remarkably similar integrin expression profiles, and share overexpression of *ITGAV*, *ITGA2*, *ITGA11*, *ITGB4*, and *ITGB5*. Also, both share underexpression of *ITGB2*, *ITG10*, *ITGA9*, *ITGA8*, *ITGA5*, *ITGA1*, *ITGAM*, *ITGAL*, and *ITGAE* (Figure 2B).

The computational analysis (Figure 8A) and immunohistochemical data (Figure 7, red color) yielded no viable heterodimer options for several cancer types, including BLCA (bladder urothelial carcinoma). BLCA, in this study, was found to possess no actionable receptors primarily through scoring via the Metric and FPKM filtering. However, as indicated earler, overexpression of both subunits in a heterodimeric pair may not be needed for a viable drug target; overexpression of one subunit with normal expression of the obligated partnering subunit may suffice. Using this relaxed criteria, three plausible targets emerge for BLCA: α3β1, αvb6, and α6b4 (Figure 8A).

While predictions based solely on transcriptome profiling data can be limited due to varying concordance between RNA and protein levels [54, 55], we attempted to increase the dependability of our scheme by integrating protein-level data. The immunohistochemistry data (Figure 6) seemingly preferentially recognized many targets for lung, liver, and cervical cancer that were unsupported by computational analysis of the RNA-Seq transcriptome data. Two potential reasons can explain this phenomenon: *1)* RNA expression does not always correspond to protein expression, and *2)* non-TCGA samples were used. Comparing non-TCGA data (IHC data) to TCGA samples (computational data) can lead to discrepancies in analysis simply because a different data source was used. It is curious that there are inconsistencies with the immunohistochemistry data. For example, when staining ITGβ4, antibodies HPA036348 and HPA036349 showed reliable results whereas antibodies CAB002422 and CAB005258 do not stain normal glands that should typically be positive for ITGβ4. In order to combat this source of inconsistency and be stringent in the criteria for IHC analysis, only validated antibodies supported by immunoblotting evidence were used (Supplementary Table 3B). A portion of this inconsistency may stem from the heterogeinety within the tissue samples stained.

The level of mutation and type of mutation are all factors to be considered in designing therapeutic ligands. Mutations could potentially affect the activation state of the integrin or alter the binding pocket of integrin. Mutated integrin subunits may lower the binding affinity of the integrin to the therapeutic ligands as the ligand was designed for wild-type integrins. This renders the tumor resistant to such treatment. In this study, we have ignored integrin mutation in our selection criteria because, in general, mutation rate of the integrin subunits for the 17 surveyed cancers were low ranging from 0.2% to 8.74%. This low level of mutation indicates that overall mutation rate was not an important factor in assessing drug targets.

In the era of precision medicine, by selecting integrins that are overexpressed in specific tumors, more effective therapeutics can be designed. Surveying the integrin expression profile of a tumor, the most overexpressed integrins can be targeted, provided that high affinity and high specificity antagonist or ligands for drug delivery against these integrin heterodimers are available. Therapeutics developed against these overexpressed integrin are intended to be short-term treatment options. The expression differences between cancer and normal tissue are high enough to minimize non-relevant effects. Furthermore, these overexpressed integrin targets can also serve as imaging targets [14]. One efficient approach to discover such ligands is to use the enabling OBOC combinatorial technology [11–14].

Many solid tumors, such as GBM, LIHC, and PAAD exhibit marked cellular and molecular heterogeneity. This is critical to consider when analyzing transcriptome data as certain focal areas in the tumor (*e.g.*, cell sub-populations) may exhibit overexpression of a marker while adjacent sections may not express the marker. Moreover, consideration of intra-tumor heterogeneity is critical in the context of drug development and tumor targeting, since the overall drug responses can be influenced by the target being expressed in a generalized manner throughout the tumor or in a more limited, focal area. In this regard, single cell analyses, spatial transcriptomics, and immunohistochemistry can be vital in discerning these patterns of expression.

In this study, we comprehensively screened the TCGA database and identified integrin expression and potential targets across 17 cancer types. Furthermore, a new method for selection of integrin-based therapeutic targets has been described (Figure 1). While this study identified actionable targets broadly for each cancer type, applying this for individual patients will be essential for achieving goals of precision medicine. In addition, while this is a bioinformatics-based analysis, it will be essential to perform further studies aimed at empirical validation of this target selection approach.

## MATERIALS AND METHODS

### Data acquisition from TCGA (the cancer genome atlas)

Non-normalized, raw counts data from The Cancer Genome Atlas was downloaded from the TCGA using the R package TCGAbiolinks [56]. All Illumina RNA-seq gene expression data for all 30 available cancer types was downloaded.

### Differential expression analysis

Gene symbols were converted from ENSEMBL ID to HGNC ID for all 30 datasets. Differential analysis was performed by analyzing expression differences between all cancer samples compared to all normal samples of a particular tumor type. Datasets with normal samples were considered, and all datasets without normal samples were discarded, since tumor-normal comparisons would not be possible. Seven datasets (ACC, DLBC, MESO, UCS, OV, TGCT, and LGG) had only tumor samples. Abbreviations of cancer types used throughout this paper are in concordance with TCGA abbreviations and the full name of the cancer type can be found in the legend to Figure 2A.

The data for the 27 integrin subunits (18 α, 9 β) was extracted from the entire RNA-seq expression datasets. The DESeq2 R package was utilized for differential expression analysis [57]. As the samples analyzed were processed at different labs worldwide and processed at different times, GC-content and length bias varied. To account for these gene-dependent dependencies, a normalization factor matrix with the same dimensions as the counts matrix was created. The normalization matrix was structured such that the row-wise geometric mean was 1, to ensure that the normalized counts for a gene was close to the mean of the unnormalized counts. Then, dispersions were estimated and the negative binomial Wald test was performed to generate results.

Results for each cancer type included, for each of the 27 integrin subunit genes, a log 2 value of expression difference between cancer and normal, a *p*-value, and a false discovery rate (FDR) adjusted according to the Benjamini-Hochberg method (Supplementary Table 2).

Log 2 value of differential expression, indicates that a value of zero, is an expression difference of 2°, or 1. Hence, a log 2 value of zero means that there exists no expression difference between cancer and normal samples. Positive log 2 values indicate increased expression in cancer samples compared to normal whereas negative indicates decreased expression in cancer samples compared to normal.

### Ranking overexpressed genes as drug targets

A scoring system was established to determine which genes in a specific cancer are considered the best potential drug targets. A mathematical formula was created that computed a metric for ranking potential drug targets.

$$\mathrm{Metric}=\left(\log\;{\mathrm{val}}_{\mathrm{gene}}\right)-\frac{FDR}{0.05}$$

*logval_gene_* = log 2 value of differential expression generated by DESeq2

*FDR* = False Discovery Rate, calculated by negative binomial model of DESeq2

The expression concerning *FDR:* $-\frac{FDR}{0.05}$ exists such that False Discovery Rates less than the accepted threshold of 0.05, keeps the negative value low whereas FDRs above the threshold severely raise the negative value. Through the construction of the formula, the best drug targets all have FDRs < 0.05, and high logarithmic change values.

### Immunohistochemistry data analysis

Immunohistochemistry (IHC) data was sourced from the Human Protein Atlas [58–60]. Using the antibody information provided by the Protein Atlas, only validated antibodies were used. If the Protein Atlas only used one antibody for their data, the antibody was used but its validity was mentioned in the discussion. The antibodies that were used for IHC analysis is depicted in Supplementary Table 3B. Both normal and tumor samples were stained according to the Protein Atlas described methods [56–58]. For each cancer, for each integrin, the Protein Atlas assigned a single qualitative value to the normal IHC level (either not detected, low, medium, or high). A segmented bar graph, showing a single integrin’s IHC values for a particular cancer type, provided the staining intensity using qualitative descriptors (not detected, low, medium, or high). Supplementary Table 3B also shows the number of samples for each tumor type. For example, the segmented bar graph charted the number of samples that showed low expression, medium expression, and so forth. Overexpression of an integrin in a cancer compared to normal was denoted when the weighted average of the staining intensity of the tumor samples was greater than the normal value assigned to the respective tumor type. The results of the overexpression can be seen in Figure 6.

In order to begin comparing IHC expression data, the qualitative descriptors were converted into quantitative values. Each descriptor for expression was paired with a value (not detected = 0, low = 0.33, medium = 0.66, and high = 1). A weighted average was calculated according to the equation shown below.

$$\left(\#\text{of high samples}\;\text{x}1\right)+\left(\#\text{of low samples x}0.33\right)+\frac{\left(\#\text{of medium samples x}0.66\right)+\left(\#\text{of not detected samples x}0\right)}{\text{Total}\#\text{of Samples}}$$

### Weighted average

A weighted average for each integrin for each tumor type was generated using only the results from validated antibodies (Supplementary Table 3A). Overexpression of an integrin subunit for a particular tumor type was determined by comparing the weighted average to the normal value. If the weighted average value was greater than the normal value, the integrin was considered to be overexpressed in that tumor type.

### Filtering integrin gene targets on FPKM expression levels

FPKM absolute expression values were obtained from the TCGA dataset analyses on The Protein Atlas. For the subunits most highly ranked according to the metric, FPKM values were used to filter this set of integrin targets further. A cut-off of 10 FPKM was employed, as that threshold defines the moderately expressed transcript.

### Determining protein heterodimers from scored genes

Since integrin subunits associate as heterodimers to assemble a productive receptor, the top genes ranked by the metric were used to predict the protein heterodimer drug targets. Rules for integrin α and β subunit association were used as summarized in Figure 2A.

## SUPPLEMENTARY MATERIALS FIGURE AND TABLES





## Abbreviations

CSC: cancer stem cell
ECM: extracellular matrix
IMD: integrin-mediated death
MMPs: metalloproteinases
PD-L1: programmed cell death ligand-1
RNA-Seq: RNA-Sequencing
FPKM: Fragments Per Kilobase of Transcript per Million mapped reads
FDR: false discovery rate
TCGA: The Cancer Genome Atlas
DE: differential expression
DEG: differentially expressed genes
IHC: immunohistochemistry
KIRP: kidney renal papillary cell carcinoma
THCA: thyroid carcinoma
READ: rectum adenocarcinoma
PRAD: prostate adenocarcinoma
LUSC: lung squamous cell carcinoma
LUAD: lung adenocarcinoma
LIHC: liver hepatocellular carcinoma
KIRC: kidney renal cell carcinoma
HNSC: head and neck squamous cell carcinoma
GBM: glioblastoma multiforme
CHOL: cholangiocarcinoma
CESC: cervical squamous cell carcinoma and endocervical adenocarcinoma
BRCA: breast invasive carcinoma
BLCA: bladder urothelial carcinoma
KICH: kidney chromophobe
PAAD: pancreatic adenocarcinoma
PCPG: paraganglioma and pheochromocytoma
