# Supplementary material for: Identification of integrin drug targets for 17 solid tumor types
Source: Oncotarget. 2018 Jul 10;9(53):30146–62. doi: 10.18632/oncotarget.25731 (PMC6059022; doi:10.18632/oncotarget.25731)
Supplement: Supplementary file 1 [file oncotarget-09-30146-s001.pdf]

Identification of integrin drug targets for 17 solid tumor types

SUPPLEMENTARY MATERIALS

Supplementary Table 1: The full name for the abbreviated cancer names used throughout this paper is outlined

4 Letter Code	Full Name	Tumor Samples	Normal Samples
KIRP	Kidney Renal Papillary Cell Carcinoma	289	32
STAD	Stomach Adenocarcinoma	481	40
READ	Rectum Adenocarcinoma	167	10
PRAD	Prostate Adenocarcinoma	499	52
LUSC	Lung Squamous Cell Carcinoma	502	49
LUAD	Lung Adenocarcinoma	535	59
LIHC	Liver Hepatocellular Carcinoma	374	50
KIRC	Kidney Renal Cell Carcinoma	539	72
HNSC	Head and Neck Squamous Cell Carcinoma	502	44
GBM	Glioblastoma Multiforme	169	5
CHOL	Cholangiocarcinoma	36	9
CESC	Cervical Squamous Cell Carcinoma and Endocervical Adenocarcinoma	306	3
BRCA	Breast Invasive Carcinoma	1109	113
BLCA	Bladder Urothelial Carcinoma	414	19
KICH	Kidney Chromophobe	65	24
PAAD	Pancreatic Adenocarcinoma	178	4
PCPG	Paraganglioma and Pheochromocytoma	183	3

Supplementary Table 2: The entire datasets used for analysis of differential expression is shown. See Supplementary_Table_2

Supplementary Table 3: The data used for immunohistochemistry analysis is depicted

A

	Breast	Cervical	Colorectal	Glioma	Head and Neck	Liver	Lung	Pancreatic	Prostate	Renal	Stomach	Urothelial
ITGAD	0.055, 0	0.0275, 0	0.0275, 0	0, 0	0.165, 0	0.0275, 0	0.06, 0	0.11, 0	0, 0	0, 0	0, 0	0.03, 0
ITGAE	0, 0	0, 0	0, 0	0, 0	0, 0	0, 0	0.03, 0	0.03, 0	0, 0	0.055, 0	0, 0	0, 0
ITGAL	0, 0	0, 0	0, 0	0, 0	0 , 0	0, 0	0, 0	0, 0	0, 0	0, 0	0, 0	0, 0
ITGAM	0, 0	0.0275, 0	0, 0	0, 0	0, 0.03	0, 0	0, 0	0, 0	0, 0	0, 0	0, 0	0, 0
ITGAV	0.496, 0.66	0.523, 0.33	0.36, 0.66	0.718, 0.33	0.832, 0.66	0.391, 0.33	0.468, 0.33	0.55, 0.66	0.462, 0.66	0.607, 1	0.385, 0.66	0.815, 1
ITGAX	0, 0	0, 0	0, 0	0, 0	0 , 0	0, 0	0, 0	0, 0	0, 0	0, 0	0, 0	0, 0
ITGA1	0.697, 1	0.275, 0.66	0.468, 0.66	0.54, 0.66	0.55, 0.66	0.495, 0.33	0.38, 0.66	0.691, 0.66	0.073, 0.33	0.288, 0.66	0.471, 0.66	0.561, 0.66
ITGA2	0.18, 1	0.33, 0.66	0.753, 1	0.165, 0	0.663, 0.66	0.468, 0.33	0.398, 0.66	0.695, 0.33	0.099 0.33	0.11, 1	0.33, 0.66	0.725, 1
ITGA2B	0, 0	0, 0	0, 0	0, 0	0 , 0	0.01375, 0	0, 0	0, 0	0, 0	0, 0	0, 0	0, 0
ITGA3	0.275, 0.33	0.581, 0.33	0.486, 0.66	0.137, 0	0.58, 0.33	0.424, 0.66	0.663, 0.66	0.816, 0.33	0.099, 0.66	0.689, 0.66	0.635, 0.66	0.86, 0.66
ITGA4	DATA NOT REPORTED											
ITGA5	0, 0	0.014, 0	0.015, 0	0, 0	0.165, 0	0.055, 0.33	0, 0	0.03, 0	0.014, 0	0, 0	0.015, 0	0.096, 0
ITGA6	0.03, 0.33	0.193, 0.33	0.595, 0.66	0, 0	0.44, 0.33	0.165, 0.33	0.12, 0.33	0.39, 0.33	0.33, 0.33	0.083, 0.66	0.429, 0.66	0.24, 1
ITGA7	0.13, 0.33	0.09, 0.66	0.1375, 0.66	0, 0.66	0.33, 0.66	0.11, 0.33	0.0925, 0.66	0.05, 0.33	0.18, 0	0.0275, 0.66	0.358, 0.66	0.11, 0.66
ITGA8	0, 0	0, 0	0, 0	0, 0	0 , 0	0, 0	0, 0	0, 0	0, 0	0, 0	0, 0	0, 0
ITGA9	0.1375, 0.33	0.1925, 0.33	0.0275, 0.33	0, 0	0, 0.66	0.165, 0.33	0.0275, 0.33	0.055, 0.33	0, 0	0.405,1	0, 0.66	0.303, 0.66
ITGA10	DATA NOT REPORTED											
ITGA11	0.18, 0.66	0.637, 0.66	0.523, 0.66	0.24, 0	0.748, 1	0.385, 0.33	0.48, 0.66	0.495, 0.33	0.099, 0.66	0.24, 0.66	0.55, 0.66	0.803, 0.66
ITGBL1	0.358, 0.66	0.248, 0.33	0.468, 0.66	0.3, 0	0.412, 0	0.193, 0	0.331, 0.33	0.578, 1	0.36, 0.33	0.055, 0.33	0.552, 0.66	0.386, 0.33
ITGB1	0.774, 1	0.814, 0.66	0.754, 1	0.275, 0.33	0.915, 0.66	0.972, 1	0.887, 0.66	0.915, 1	0.662, 0.66	0.915, 0.33	0.723, 0.66	1, 1
ITGB2	0, 0	0, 0	0, 0	0.249, 0.165	0 , 0	0, 0	0, 0	0, 0	0.031, 0	0, 0	0, 0	0, 0
ITGB3	0, 0	0, 0	0, 0	0, 0	0 , 0	0.33, 0	0, 0	0.033, 0	0.0275, 0	0.523, 0	0, 0	0, 0
ITGB4	0.255, 0.33	0.539, 0.465	0.517, 0.66	0.21, 0	0.538, 0.33	0.445, 0.33	0.456, 0.33	0.377, 0	0.275, 0.33	0.3, 0	0.409, 0.66	0.431, 0.66
ITGB5	0.55, 0.66	0.33, 0.165	0.45, 0.66	0.633, 0.33	0.663, 0.33	0.563, 0.66	0.413, 0.33	0.396, 0.33	0.757, 0.33	0.297, 0.66	0.33, 0.66	0.551, 0.33
ITGB6	0.442, 0	0.647, 0	0.59, 0.66	0.029, 0	0.247, 0	0.414, 0	0.461, 0.33	0.529, 0.33	0.571, 0	0.286, 0.33	0.442, 0.33	0.552, 1
ITGB7	0.916, 1	0.773, 0.66	0.966, 0.66	0.495, 0.33	0.915, 0.66	0.938, 1	0.815, 1	0.898, 1	0.915, 1	0.915, 0.66	0.845, 1	0.876, 1
ITGB8	0.413, 0.33	0.22, 0.33	0.578, 0.5	0.11, 0.33	0, 0.66	0.357, 0	0.193, 0.165	0.12, 0.66	0.523, 0.33	0.09, 0.66	0.11, 0.66	0.248, 0.66

B

	Antibodies	Breast	Cervical	Colorectal	Glioma	Head and Neck	Liver	Lung	Pancreatic	Prostate	Renal	Stomach	Urothelial
ITGAD	HPA026786	12	12	12	12	4	12	11	12	12	12	11	11
ITGAE	HPA036313	11	11	12	11	3	11	11	11	10	12	11	11
ITGAL	CAB025011	11	12	12	11	4	12	11	12	12	12	12	11
ITGAM	HPA002274	12	12	12	12	4	12	12	12	12	12	11	12
ITGAV	CAB002499	12	12	11	12	4	12	12	9	10	12	12	11
ITGAX	CAB004458	12	10	11	12	3	11	11	12	10	12	11	12
ITGA1	HPA042555	11	12	12	11	3	12	11	12	9	10	7	10
ITGA2	CAB017690	11	10	11	12	4	12	10	10	10	12	10	11
ITGA2B	HPA0311168, HPA031170	21	22	23	23	8	24	22	20	21	24	20	22
ITGA3	HPA008572	12	12	12	12	4	11	12	11	10	12	12	12
ITGA4	Data Not Reported												
ITGA5	CAB009008, HPA002642	24	23	21	24	8	24	24	22	23	23	22	24
ITGA6	HPA012696	11	12	10	12	2	10	11	11	10	12	10	11
ITGA7	HPA008427	11	11	12	12	4	12	12	12	11	12	12	12
ITGA8	HPA003432	12	11	9	11	4	12	12	12	11	11	10	12
ITGA9	HPA030609	12	12	12	12	4	12	12	12	11	12	12	12
ITGA10	Data Not Reported												

ITGA11	HPA051813	11	12	12	11	4	12	11	10	10	11	12	12
ITGBL1	HPA005676	12	12	12	11	4	12	12	12	11	12	12	12
ITGB1	CAB003434	12	11	11	12	4	12	12	12	10	12	11	12
ITGB2	HPA008877, HPA016894	24	24	24	23	8	23	23	21	22	24	24	22
ITGB3	HPA027852	12	12	11	12	4	12	12	12	10	12	12	11
ITGB4	HPA036348, HPA036349	22	19	23	22	8	24	22	21	18	24	21	23
ITGB5	HPA001820	10	12	12	9	3	8	11	10	11	12	11	10
ITGB6	HPA023626, CAB073538	24	22	22	23	8	24	23	20	22	24	21	24
ITGB7	HPA042277	12	12	10	10	4	11	11	10	12	10	11	11
ITGB8	HPA027796	12	12	12	12	4	12	12	11	12	11	12	11

A

	BLCA	BRCA	CESC	CHOL	GBM	HNSC	KICH	KIRC
ITGAD	0	0.48	0	1.55	0	0	2.83	4.58
ITGAE	0.54	0	0	2.24	0	0.39	-0.87	0
ITGAL	0	1.04	0	0	1.43	0.75	0	2.63
ITGAM	0	0.48	0	3.45	1.43	0	0	1.8
ITGAV	0	0	0	3.36	0.93	1.23	0	-0.43
ITGAX	0	0.89	1.84	1.75	0	1.46	1.41	3.33
ITGA1	-1.86	-1.05	0	0	2.86	1.54	0	0.77
ITGA2	0.87	-0.46	2.84	6.25	0	0	-0.8	-1
ITGA2B	1.69	0.72	0	3.13	0	1.83	-1.34	0
ITGA3	1.11	-0.27	0	5.3	0	1.83	-1.57	0.48
ITGA4	0	0.57	0	1.14	3.2	1.04	-0.61	1.62
ITGA5	-1.88	0.27	0	2.45	2.95	2.53	0	1.88
ITGA6	0	-0.85	0	2.51	0	2.01	1.53	0.33
ITGA7	-2.47	-3.39	-3.37	0	1.58	-1.2	0.68	0.83
ITGA8	-3.01	-0.58	-2.84	1.56	0	0	-2.47	-0.44
ITGA9	-1.63	-1.09	-3.41	0	-1.17	-0.88	-0.7	0
ITGA10	0	-0.88	0	1.89	0	0	-2.14	0.95
ITGA11	0	1.08	-4.55	2.59	0	0	-2.01	0
ITGBL1	0	0.69	0	2.39	1.92	0.87	-1.81	0
ITGB1	0	-0.8	0	2.71	1.66	0.74	-1.25	0.27
ITGB2	0	0.75	0	1.53	1.78	0.97	0	2.37
ITGB3	-1.83	0	0	0	2.83	0	-1.34	-0.89
ITGB4	1.01	-0.47	2.33	5.88	1.61	1.4	-1.65	0.52
ITGB5	0.6	0.27	0	2.32	0	0.78	-0.97	-0.35
ITGB6	1.12	0	5.91	6.14	1.21	1.39	-2.73	-2.81
ITGB7	0	0.76	2.57	1.27	0	0	0.89	0.58
ITGB8	0	-0.62	0	4.55	1.24	0	-1.92	-0.53

B

	KIRP	LIHC	LUAD	LUSC	PAAD	PCPG	PRAD	READ	STAD
ITGAD	0	0	0	-1.07	-3.53	0	1.23	-1.94	1.65
ITGAE	0	0	-0.31	-0.56	0	0	0	0	0.78
ITGAL	1.05	0.37	-0.71	-2.1	-2.68	0	0	-1.31	0.8
ITGAM	1.58	0.87	-0.57	-1.96	0	0	-0.38	0	1.22
ITGAV	-1.09	1.42	1.2	0.81	0	0	0	0	0.68
ITGAX	3.02	0.62	-0.61	-1.67	-1.72	0	0.68	0	2.36
ITGA1	-1.38	0	-0.37	-2.19	0	-1.59	-1.23	-1.32	-0.7
ITGA2	-1.36	2.32	1.79	1.21	0	-2.65	-1.48	0	1.82
ITGA2B	0	0	0	-1.08	0	0	-0.66	0	0
ITGA3	1.38	0	0.7	-0.78	0	0	-0.91	0	0.86
ITGA4	0	0	0	-0.91	0	2.35	0	-1.39	1.01
ITGA5	0	1.15	-0.47	-0.7	0	0	-1.27	-1.33	0
ITGA6	-0.44	2.02	0	1.36	1.45	2.76	-0.62	0	1.44
ITGA7	0	1.1	0.34	-1.42	0	0	-1.15	-2.44	-1.12
ITGA8	-2.88	0	-1.81	-3.66	0	0	-1.25	-1.69	-1.84
ITGA9	-1.89	-1.41	-0.37	-2.38	0	-2.55	-1.4	0	-0.96
ITGA10	-2.1	1.36	-0.57	-2.59	0	0	0.38	0	0
ITGA11	-1.45	1.75	3.1	1.88	0	0	0	1.69	2.22
ITGBL1	0	0.82	0.5	-1.25	0	0	1.29	1.89	1.58
ITGB1	0	0.59	0.49	-0.44	0	0	-0.98	0	0.63
ITGB2	1.55	0	-0.42	-1.47	-1.68	0	0	0	1.35
ITGB3	0	0	0	-1.06	0	3.62	-1.5	-1.73	0
ITGB4	1.04	2.07	2.03	2.57	1.74	0	-1.31	0	1.4
ITGB5	-0.84	0.89	0.69	0.52	0	0	0	0	0.67
ITGB6	0	0	0	-1.17	0	0	-1.77	0	1.59
ITGB7	0	0.46	0.76	-0.58	0	0	-0.53	-1.26	0
ITGB8	1.63	0	1.67	2.14	0	0	-1.29	0.91	1.33

Supplementary Figure 1: Log 2 fold change values for each integrin across all surveyed cancer types shown. Zero values indicate that the false discovery rate was greater than the accepted 0.05. The green highlighted cells represent genes that are overexpressed in cancer compared to normal samples.
