# Supplementary material for: Identification of integrin drug targets for 17 solid tumor types
Source: Oncotarget. 2018 Jul 10;9(53):30146–62. doi: 10.18632/oncotarget.25731 (PMC6059022; doi:10.18632/oncotarget.25731)
Supplement: Supplementary file 2 [file oncotarget-09-30146-s002.docx]

| **GENE SYMBOL** | **log2FoldChange** | **pvalue** | **padj** |
| --- | --- | --- | --- |
|  |  | **BLCA** |  |
| ITGAD | 1.604394845 | #N/A | #N/A |
| ITGAE | 0.537319866 | 0.010807844 | 0.028820918 |
| ITGAL | -0.521974859 | 0.18878185 | 0.302050961 |
| ITGAM | 0.331546274 | 0.402803367 | 0.460346705 |
| ITGAV | 0.271379538 | 0.287248164 | 0.382997551 |
| ITGAX | 0.618496827 | 0.075732435 | 0.127549364 |
| ITGA1 | -1.857289448 | 1.12723E-10 | 1.20238E-09 |
| ITGA2 | 0.869398328 | 0.020536256 | 0.046940013 |
| ITGA2B | 1.685268937 | 4.50649E-05 | 0.000206011 |
| ITGA3 | 1.111079576 | 0.001178069 | 0.004514007 |
| ITGA4 | 0.155678782 | 0.684458955 | 0.706538276 |
| ITGA5 | -1.877088146 | 9.42418E-08 | 7.53935E-07 |
| ITGA6 | -0.210176593 | 0.556697494 | 0.59381066 |
| ITGA7 | -2.471792069 | 3.05424E-11 | 4.88678E-10 |
| ITGA8 | -3.01254329 | 2.66455E-18 | 8.52655E-17 |
| ITGA9 | -1.634931483 | 2.17851E-07 | 1.39425E-06 |
| ITGA10 | -0.573386225 | 0.058133813 | 0.103349 |
| ITGA11 | 0.807354423 | 0.055196291 | 0.103349 |
| ITGBL1 | -0.273315195 | 0.546119647 | 0.59381066 |
| ITGB1 | -0.33035608 | 0.230634095 | 0.320882219 |
| ITGB2 | 0.33632137 | 0.399151623 | 0.460346705 |
| ITGB3 | -1.825041743 | 1.48511E-05 | 7.92057E-05 |
| ITGB4 | 1.017676371 | 0.001269565 | 0.004514007 |
| ITGB5 | 0.591613863 | 0.023831523 | 0.050840583 |
| ITGB6 | 1.111458734 | 0.003720364 | 0.011905165 |
| ITGB7 | -0.080942448 | 0.80008898 | 0.80008898 |
| ITGB8 | 0.482732185 | 0.223872023 | 0.320882219 |

| **GENE SYMBOL** | **log2FoldChange** | **pvalue** | **padj** |
| --- | --- | --- | --- |
|  |  | **CHOL** |  |
| ITGAD | 1.545365641 | 0.029594435 | 0.037486285 |
| ITGAE | 2.240217276 | 4.23669E-18 | 2.68324E-17 |
| ITGAL | 0.272088685 | 0.607042522 | 0.640767106 |
| ITGAM | 3.455672933 | 9.04371E-16 | 3.81846E-15 |
| ITGAV | 3.368887404 | 8.91145E-18 | 4.83764E-17 |
| ITGAX | 1.751255036 | 1.2222E-05 | 2.32219E-05 |
| ITGA1 | -0.607311523 | 0.068459096 | 0.078831686 |
| ITGA2 | 6.253373001 | 8.37023E-29 | 1.59034E-27 |
| ITGA2B | 3.136105883 | 4.50755E-10 | 1.14191E-09 |
| ITGA3 | 5.300663016 | 3.8823E-22 | 3.68818E-21 |
| ITGA4 | 1.141789753 | 0.007473416 | 0.01051814 |
| ITGA5 | 2.450314425 | 3.96061E-09 | 9.40644E-09 |
| ITGA6 | 2.513304355 | 9.18314E-14 | 3.48959E-13 |
| ITGA7 | 0.797149828 | 0.044026068 | 0.053967438 |
| ITGA8 | 1.56705116 | 0.000217178 | 0.000343866 |
| ITGA9 | -0.366201728 | 0.390692822 | 0.424180778 |
| ITGA10 | 1.892169472 | 1.75408E-05 | 3.17404E-05 |
| ITGA11 | 2.599254309 | 3.01402E-08 | 6.73722E-08 |
| ITGBL1 | 2.392715901 | 0.000131238 | 0.000226684 |
| ITGB1 | 2.710695106 | 2.24841E-10 | 6.10283E-10 |
| ITGB2 | 1.825908265 | 0.000331804 | 0.000504342 |
| ITGB3 | 0.935941529 | 0.079526169 | 0.088882189 |
| ITGB4 | 5.888175915 | 2.02909E-37 | 7.71053E-36 |
| ITGB5 | 2.32588543 | 2.51354E-11 | 7.95956E-11 |
| ITGB6 | 6.164798698 | 4.14315E-13 | 1.43127E-12 |
| ITGB7 | 1.270513791 | 0.020015142 | 0.026226738 |
| ITGB8 | 4.559346672 | 1.08016E-17 | 5.13075E-17 |

| **GENE SYMBOL** | **log2FoldChange** | **pvalue** | **padj** |
| --- | --- | --- | --- |
|  |  | **GBM** |  |
| ITGAD | 1.434252286 | #N/A | #N/A |
| ITGAE | 0.663813104 | 0.086935303 | 0.120371958 |
| ITGAL | 1.434916065 | 0.004291446 | 0.011035147 |
| ITGAM | 1.439461285 | 0.004212285 | 0.011035147 |
| ITGAV | 0.93985349 | 0.019881826 | 0.037670828 |
| ITGAX | 0.491844304 | 0.344210401 | 0.402770544 |
| ITGA1 | 2.869497799 | 1.30796E-10 | 4.70865E-09 |
| ITGA2 | 0.907955747 | 0.09906178 | 0.132082373 |
| ITGA2B | -0.744817798 | 0.150681841 | 0.193733795 |
| ITGA3 | 1.275896358 | 0.052684116 | 0.079046604 |
| ITGA4 | 3.205930249 | 3.60092E-09 | 4.32111E-08 |
| ITGA5 | 2.954762527 | 8.20546E-10 | 1.47698E-08 |
| ITGA6 | 0.791852919 | 0.059553073 | 0.085756425 |
| ITGA7 | 1.5820496 | 0.003378902 | 0.010136707 |
| ITGA8 | 0.273637058 | 0.644710969 | 0.663131282 |
| ITGA9 | -1.175718062 | 0.014948002 | 0.03007927 |
| ITGA10 | 1.850666597 | #N/A | #N/A |
| ITGA11 | 1.439287116 | #N/A | #N/A |
| ITGBL1 | 1.925984286 | 0.00808415 | 0.019401959 |
| ITGB1 | 1.661040695 | 0.000114398 | 0.000514789 |
| ITGB2 | 1.782237134 | 0.00022568 | 0.00090272 |
| ITGB3 | 2.831269949 | 2.2659E-06 | 1.35954E-05 |
| ITGB4 | 1.13926853 | 0.052697736 | 0.079046604 |
| ITGB5 | 1.617778446 | 0.000610237 | 0.001997139 |
| ITGB6 | 1.655958817 | 0.039995672 | 0.068564009 |
| ITGB7 | 1.217152139 | 0.021056231 | 0.037901217 |
| ITGB8 | 1.241003348 | 0.015039635 | 0.03007927 |

| **GENE SYMBOL** | **log2FoldChange** | **pvalue** | **padj** |
| --- | --- | --- | --- |
|  |  | **HNSC** |  |
| ITGAD | 1.60724381 | #N/A | #N/A |
| ITGAE | 0.396275431 | 0.006360816 | 0.010601361 |
| ITGAL | 0.755060402 | 0.001433108 | 0.002639937 |
| ITGAM | 1.463132365 | #N/A | #N/A |
| ITGAV | 1.231500094 | 1.93146E-12 | 6.7601E-12 |
| ITGAX | 1.469862609 | 2.91618E-13 | 1.13407E-12 |
| ITGA1 | 1.543835578 | 2.88459E-17 | 1.44229E-16 |
| ITGA2 | 0.268721266 | 0.143117442 | 0.200364419 |
| ITGA2B | 1.836234343 | 1.15913E-16 | 5.07117E-16 |
| ITGA3 | 1.838230221 | 1.36621E-20 | 1.19543E-19 |
| ITGA4 | 1.049968008 | 9.9538E-06 | 2.28846E-05 |
| ITGA5 | 2.537568028 | 1.8858E-36 | 6.6003E-35 |
| ITGA6 | 2.012261882 | 1.19568E-23 | 2.09244E-22 |
| ITGA7 | -1.201441279 | 7.71028E-07 | 1.92757E-06 |
| ITGA8 | -0.258376233 | 0.227234117 | 0.284042646 |
| ITGA9 | -0.882502606 | 1.99748E-05 | 4.11246E-05 |
| ITGA10 | -0.155261382 | 0.422138569 | 0.492494997 |
| ITGA11 | 2.251290779 | #N/A | #N/A |
| ITGBL1 | 0.87806832 | 0.00517479 | 0.009055882 |
| ITGB1 | 0.743035604 | 6.61846E-05 | 0.000128692 |
| ITGB2 | 0.970861472 | 1.04615E-05 | 2.28846E-05 |
| ITGB3 | -0.139345815 | 0.581112972 | 0.656095291 |
| ITGB4 | 1.403147725 | 2.65483E-17 | 1.44229E-16 |
| ITGB5 | 0.782722364 | 4.46597E-08 | 1.30257E-07 |
| ITGB6 | 1.396241285 | 4.39017E-10 | 1.39687E-09 |
| ITGB7 | 0.097485942 | 0.648733385 | 0.70955214 |
| ITGB8 | 0.344825292 | 0.06428598 | 0.097826491 |

| **GENE SYMBOL** | **log2FoldChange** | **pvalue** | **padj** |
| --- | --- | --- | --- |
|  |  | **KICH** |  |
| ITGAD | 2.834518208 | 3.30451E-12 | 1.6296E-11 |
| ITGAE | -0.876378152 | 4.72496E-06 | 1.08396E-05 |
| ITGAL | -0.24577375 | 0.40325122 | 0.453169587 |
| ITGAM | -0.336778843 | 0.328211296 | 0.387886078 |
| ITGAV | -0.253044587 | 0.302276292 | 0.368399231 |
| ITGAX | 1.410344063 | 1.18196E-05 | 2.42613E-05 |
| ITGA1 | -0.439941426 | 0.063299787 | 0.08816756 |
| ITGA2 | -0.805677811 | 0.008605595 | 0.013424728 |
| ITGA2B | -1.349672182 | 4.36508E-06 | 1.06399E-05 |
| ITGA3 | -1.570563834 | 1.04655E-07 | 3.13966E-07 |
| ITGA4 | -0.610733804 | 0.032559689 | 0.047030662 |
| ITGA5 | -0.43182891 | 0.086695647 | 0.116590697 |
| ITGA6 | 1.537824704 | 9.1474E-10 | 3.8558E-09 |
| ITGA7 | 0.687317203 | 0.015312139 | 0.022968209 |
| ITGA8 | -2.474616335 | 4.63659E-23 | 4.52068E-22 |
| ITGA9 | -0.70058771 | 0.001846017 | 0.003130202 |
| ITGA10 | -2.14898339 | 1.38014E-09 | 4.89323E-09 |
| ITGA11 | -2.005517266 | 9.88667E-10 | 3.8558E-09 |
| ITGBL1 | -1.810400316 | 3.85054E-06 | 1.00114E-05 |
| ITGB1 | -1.257856915 | 2.05038E-07 | 5.71177E-07 |
| ITGB2 | 0.136358311 | 0.672065482 | 0.672065482 |
| ITGB3 | -1.341072418 | 0.000145953 | 0.000284608 |
| ITGB4 | -1.658585457 | 3.34277E-12 | 1.6296E-11 |
| ITGB5 | -0.978499119 | 5.24484E-06 | 1.13638E-05 |
| ITGB6 | -2.732951585 | 4.25196E-15 | 2.76378E-14 |
| ITGB7 | 0.899033225 | 0.000341123 | 0.000604718 |
| ITGB8 | -1.920613598 | 3.70842E-09 | 1.20524E-08 |

| **GENE SYMBOL** | **log2FoldChange** | **pvalue** | **padj** |
| --- | --- | --- | --- |
|  |  | **KIRC** |  |
| ITGAD | 4.587967114 | 8.8236E-107 | 1.6324E-105 |
| ITGAE | 0.106779795 | 0.285236324 | 0.310404234 |
| ITGAL | 2.634515687 | 8.97343E-67 | 8.30042E-66 |
| ITGAM | 1.802617636 | 6.15704E-35 | 2.53123E-34 |
| ITGAV | -0.435265541 | 0.000277625 | 0.000466915 |
| ITGAX | 3.337844753 | 3.1232E-107 | 1.1556E-105 |
| ITGA1 | 0.776769113 | 4.05736E-10 | 1.00082E-09 |
| ITGA2 | -1.000339105 | 2.81181E-13 | 7.4312E-13 |
| ITGA2B | 0.750599446 | #N/A | #N/A |
| ITGA3 | 0.482096148 | 0.000196479 | 0.000346178 |
| ITGA4 | 1.626882187 | 8.39058E-28 | 3.10451E-27 |
| ITGA5 | 1.888842588 | 1.43804E-47 | 7.60105E-47 |
| ITGA6 | 0.332641603 | 0.011443739 | 0.015122083 |
| ITGA7 | 0.837461256 | 6.03557E-10 | 1.39573E-09 |
| ITGA8 | -0.443691768 | 0.004303414 | 0.006124089 |
| ITGA9 | 0.142006948 | 0.322544827 | 0.34097596 |
| ITGA10 | 0.957742772 | 1.54947E-08 | 3.18502E-08 |
| ITGA11 | -0.345668102 | 0.052400368 | 0.062542375 |
| ITGBL1 | 0.372254668 | 0.090618787 | 0.101602882 |
| ITGB1 | 0.27765423 | 0.021052154 | 0.025964323 |
| ITGB2 | 2.370567939 | 1.18986E-62 | 8.80494E-62 |
| ITGB3 | -0.893269737 | 8.33886E-09 | 1.81493E-08 |
| ITGB4 | 0.524309064 | 0.000651697 | 0.001004699 |
| ITGB5 | -0.3517094 | 0.000440956 | 0.000709364 |
| ITGB6 | -2.815563961 | 5.57251E-43 | 2.57728E-42 |
| ITGB7 | 0.585379132 | 9.32997E-06 | 1.81689E-05 |
| ITGB8 | -0.534324601 | 0.000910634 | 0.001347738 |

| **GENE SYMBOL** | **log2FoldChange** | **pvalue** | **padj** |
| --- | --- | --- | --- |
|  |  | **KIRP** |  |
| ITGAD | 4.95019539 | #N/A | #N/A |
| ITGAE | 0.172995415 | 0.198304517 | 0.293771313 |
| ITGAL | 1.05246837 | 2.92629E-05 | 5.85258E-05 |
| ITGAM | 1.581236363 | 2.55274E-11 | 1.31284E-10 |
| ITGAV | -1.090203251 | 1.15082E-06 | 2.95926E-06 |
| ITGAX | 3.028776311 | 1.00553E-39 | 3.61989E-38 |
| ITGA1 | -1.387126373 | 6.56562E-10 | 2.36362E-09 |
| ITGA2 | -1.369897988 | 3.08109E-08 | 8.53225E-08 |
| ITGA2B | 0.015555813 | 0.954790465 | 0.954790465 |
| ITGA3 | 1.388863288 | 3.13365E-11 | 1.41014E-10 |
| ITGA4 | -0.316088118 | 0.204007856 | 0.293771313 |
| ITGA5 | -0.12506373 | 0.557289425 | 0.6079521 |
| ITGA6 | -0.446205673 | 0.01821702 | 0.031229176 |
| ITGA7 | -0.134696579 | 0.456335075 | 0.529937507 |
| ITGA8 | -2.889781543 | 5.85517E-20 | 1.05393E-18 |
| ITGA9 | -1.898319195 | 1.96269E-17 | 2.35523E-16 |
| ITGA10 | -2.109053216 | 1.41437E-13 | 1.27294E-12 |
| ITGA11 | -1.457920162 | 8.4118E-06 | 1.89265E-05 |
| ITGBL1 | -0.636901249 | 0.123293539 | 0.192981192 |
| ITGB1 | -0.211035566 | 0.337043973 | 0.43334225 |
| ITGB2 | 1.554024906 | 8.06561E-10 | 2.63965E-09 |
| ITGB3 | 0.231028031 | 0.508380763 | 0.571928358 |
| ITGB4 | 1.045625752 | 1.52994E-05 | 3.23988E-05 |
| ITGB5 | -0.849435685 | 3.4628E-06 | 8.31073E-06 |
| ITGB6 | -0.364069026 | 0.274440425 | 0.365920567 |
| ITGB7 | 0.228235553 | 0.253988953 | 0.351677011 |
| ITGB8 | 1.631583717 | 7.5951E-11 | 3.03804E-10 |

| **GENE SYMBOL** | **log2FoldChange** | **pvalue** | **padj** |
| --- | --- | --- | --- |
|  |  | **LIHC** |  |
| ITGAD | -0.439585448 | #N/A | #N/A |
| ITGAE | 1.585280187 | #N/A | #N/A |
| ITGAL | 0.377770149 | 0.031763628 | 0.043583045 |
| ITGAM | 0.871782904 | 0.000142034 | 0.000274598 |
| ITGAV | 1.428799637 | 1.77016E-12 | 7.33354E-12 |
| ITGAX | 0.626628856 | 0.001136015 | 0.002059027 |
| ITGA1 | 0.090611897 | 0.476540184 | 0.539632414 |
| ITGA2 | 2.322361989 | 4.51284E-20 | 6.54362E-19 |
| ITGA2B | 0.209872187 | 0.432982036 | 0.523186627 |
| ITGA3 | 1.858306743 | #N/A | #N/A |
| ITGA4 | -0.243894703 | 0.293424415 | 0.369969915 |
| ITGA5 | 1.153108255 | 4.56972E-12 | 1.32522E-11 |
| ITGA6 | 2.023152924 | 5.67672E-33 | 1.64625E-31 |
| ITGA7 | 1.107110636 | 2.22066E-12 | 8.04991E-12 |
| ITGA8 | 0.861488691 | #N/A | #N/A |
| ITGA9 | -1.411244276 | 3.48442E-13 | 2.02096E-12 |
| ITGA10 | 1.365274142 | 3.46047E-12 | 1.11504E-11 |
| ITGA11 | 1.753740781 | 2.41722E-14 | 1.75248E-13 |
| ITGBL1 | 0.824511629 | 0.010115566 | 0.01466757 |
| ITGB1 | 0.590248778 | 0.001641592 | 0.002800363 |
| ITGB2 | 0.01011476 | 0.960551601 | 0.993886345 |
| ITGB3 | 0.17264641 | 0.483808371 | 0.539632414 |
| ITGB4 | 2.074617354 | 5.45832E-16 | 5.27638E-15 |
| ITGB5 | 0.890984726 | 2.28945E-10 | 6.03583E-10 |
| ITGB6 | 2.181180725 | #N/A | #N/A |
| ITGB7 | 0.467786396 | 0.033063 | 0.043583045 |
| ITGB8 | 0.534318808 | #N/A | #N/A |

| **GENE SYMBOL** | **log2FoldChange** | **pvalue** | **padj** |
| --- | --- | --- | --- |
|  |  | **LUAD** |  |
| ITGAD | 0.303957863 | 0.179783889 | 0.230123378 |
| ITGAE | -0.310208471 | 0.019539918 | 0.029775114 |
| ITGAL | -0.718743327 | 9.46641E-06 | 3.02925E-05 |
| ITGAM | -0.574283599 | 0.001854256 | 0.004238299 |
| ITGAV | 1.202890398 | 3.64747E-18 | 2.33438E-17 |
| ITGAX | -0.614863322 | 0.000241757 | 0.000644685 |
| ITGA1 | -0.372715016 | 0.009440222 | 0.015899322 |
| ITGA2 | 1.798378347 | 7.68434E-20 | 6.14747E-19 |
| ITGA2B | 0.188747543 | 0.414668124 | 0.457564826 |
| ITGA3 | 0.706455124 | 0.000053239 | 0.000154877 |
| ITGA4 | 0.102197514 | 0.522138326 | 0.556947547 |
| ITGA5 | -0.475636478 | 0.005979311 | 0.011255174 |
| ITGA6 | 0.044274067 | 0.811678095 | 0.811678095 |
| ITGA7 | 0.344331346 | 0.024807316 | 0.036083369 |
| ITGA8 | -1.812405155 | 9.6759E-23 | 1.0321E-21 |
| ITGA9 | -0.374521498 | 0.029459026 | 0.040986471 |
| ITGA10 | -0.574935408 | 0.003108849 | 0.006217698 |
| ITGA11 | 3.108840904 | 1.16765E-45 | 3.73648E-44 |
| ITGBL1 | 0.509306371 | 0.008398842 | 0.014931275 |
| ITGB1 | 0.492401556 | 0.000413497 | 0.001017838 |
| ITGB2 | -0.424947026 | 0.012948743 | 0.020717988 |
| ITGB3 | 1.531622928 | #N/A | #N/A |
| ITGB4 | 2.031621986 | 1.87329E-23 | 2.99726E-22 |
| ITGB5 | 0.691232607 | 4.87645E-07 | 2.22924E-06 |
| ITGB6 | 0.287225493 | 0.128577378 | 0.171436504 |
| ITGB7 | 0.768017033 | 3.09795E-06 | 1.23918E-05 |
| ITGB8 | 1.676712108 | 5.31619E-12 | 2.8353E-11 |

| **GENE SYMBOL** | **log2FoldChange** | **pvalue** | **padj** |
| --- | --- | --- | --- |
|  |  | **LUSC** |  |
| ITGAD | -1.072976319 | 1.86531E-06 | 2.69434E-06 |
| ITGAE | -0.565981865 | 7.46937E-06 | 0.000010045 |
| ITGAL | -2.107578639 | 5.39322E-26 | 2.33706E-25 |
| ITGAM | -1.960530646 | 1.31645E-21 | 4.6674E-21 |
| ITGAV | 0.815057304 | 1.42517E-08 | 2.77909E-08 |
| ITGAX | -1.678010142 | 8.71315E-22 | 3.39813E-21 |
| ITGA1 | -2.197246507 | 8.41403E-47 | 6.56295E-46 |
| ITGA2 | 1.215108472 | 3.873E-12 | 8.39151E-12 |
| ITGA2B | -1.085258113 | 1.46377E-07 | 2.48204E-07 |
| ITGA3 | -0.781259504 | 0.000166603 | 0.000209597 |
| ITGA4 | -0.911697665 | 8.35062E-07 | 1.3027E-06 |
| ITGA5 | -0.707535937 | 3.84364E-05 | 4.99673E-05 |
| ITGA6 | 1.365693635 | 9.32686E-14 | 2.13969E-13 |
| ITGA7 | -1.429293542 | 4.45336E-16 | 1.24058E-15 |
| ITGA8 | -3.662798794 | 1.09981E-67 | 2.14462E-66 |
| ITGA9 | -2.38940928 | 9.89472E-36 | 5.51277E-35 |
| ITGA10 | -2.591151363 | 2.73634E-45 | 1.77862E-44 |
| ITGA11 | 1.880632095 | 4.18341E-15 | 1.08769E-14 |
| ITGBL1 | -1.253084236 | 5.62137E-08 | 1.04397E-07 |
| ITGB1 | -0.44681504 | 0.0018263 | 0.002094873 |
| ITGB2 | -1.475363401 | 2.86138E-14 | 6.9746E-14 |
| ITGB3 | -1.064453618 | 1.29608E-06 | 1.94411E-06 |
| ITGB4 | 2.576983806 | 1.26011E-49 | 1.2286E-48 |
| ITGB5 | 0.529686207 | 0.000310443 | 0.000378353 |
| ITGB6 | -1.173948954 | 1.68057E-07 | 2.73092E-07 |
| ITGB7 | -0.589415106 | 0.000723366 | 0.000854887 |
| ITGB8 | 2.147417211 | 1.26307E-30 | 6.15745E-30 |

| **GENE SYMBOL** | **log2FoldChange** | **pvalue** | **padj** |
| --- | --- | --- | --- |
|  |  | **PAAD** |  |
| ITGAD | -3.53699893 | 1.96906E-05 | 0.000748242 |
| ITGAE | -0.869005245 | 0.025881729 | 0.089409609 |
| ITGAL | -2.689401232 | 0.000291923 | 0.00554654 |
| ITGAM | -1.08954816 | 0.077053522 | 0.162668547 |
| ITGAV | 0.014001188 | 0.980060641 | 0.989664517 |
| ITGAX | -1.720997819 | 0.00318098 | 0.03337682 |
| ITGA1 | -0.532803286 | 0.292240249 | 0.462713727 |
| ITGA2 | 0.974802027 | 0.112367187 | 0.2033311 |
| ITGA2B | -1.209467191 | 0.033508197 | 0.097947037 |
| ITGA3 | 1.223598328 | 0.030463574 | 0.096467986 |
| ITGA4 | -1.42218212 | 0.054904535 | 0.139091488 |
| ITGA5 | 0.442340551 | 0.42626729 | 0.623006039 |
| ITGA6 | 1.44989678 | 0.010662108 | 0.050645013 |
| ITGA7 | 0.198840755 | 0.733986439 | 0.863440626 |
| ITGA8 | -0.667209515 | 0.305684481 | 0.464640411 |
| ITGA9 | -0.087656391 | 0.881758873 | 0.930745477 |
| ITGA10 | 1.123256684 | 0.095580356 | 0.181602676 |
| ITGA11 | 0.363212497 | 0.631579636 | 0.800000872 |
| ITGBL1 | 0.115141351 | 0.875454332 | 0.930745477 |
| ITGB1 | 0.152666394 | 0.776697316 | 0.868073471 |
| ITGB2 | -1.688562737 | 0.005252135 | 0.039916227 |
| ITGB3 | -0.38045984 | 0.585210176 | 0.79421381 |
| ITGB4 | 1.746885757 | 0.007734637 | 0.04198803 |
| ITGB5 | 0.937741771 | 0.049636043 | 0.134726401 |
| ITGB6 | 1.064324285 | 0.196316228 | 0.339091666 |
| ITGB7 | -1.584180072 | 0.015058092 | 0.063578612 |
| ITGB8 | 0.654541927 | 0.241201275 | 0.398506455 |

| **GENE SYMBOL** | **log2FoldChange** | **pvalue** | **padj** |
| --- | --- | --- | --- |
|  |  | **BRCA** |  |
| ITGAD | 0.479592577 | 0.002442463 | 0.003287931 |
| ITGAE | 0.07263194 | 0.369985895 | 0.404672073 |
| ITGAL | 1.037767875 | 7.8165E-17 | 3.87272E-16 |
| ITGAM | 0.476621655 | 9.58959E-05 | 0.000159826 |
| ITGAV | 0.182737705 | 0.067446409 | 0.076149172 |
| ITGAX | 0.894556149 | 1.3635E-15 | 5.30252E-15 |
| ITGA1 | -1.047469242 | 5.19002E-27 | 4.54126E-26 |
| ITGA2 | -0.459398301 | 0.000487812 | 0.000711392 |
| ITGA2B | 0.71574716 | 5.85784E-07 | 1.07908E-06 |
| ITGA3 | -0.266273378 | 0.016389688 | 0.019121303 |
| ITGA4 | 0.570666698 | 9.92777E-06 | 1.73736E-05 |
| ITGA5 | 0.270889934 | 0.003176887 | 0.004118186 |
| ITGA6 | -0.854402397 | 6.93107E-12 | 2.02156E-11 |
| ITGA7 | -3.396048729 | 1.7963E-139 | 6.2869E-138 |
| ITGA8 | -0.584615774 | 3.45459E-07 | 7.1124E-07 |
| ITGA9 | -1.088503385 | 6.29446E-21 | 4.40612E-20 |
| ITGA10 | -0.878142831 | 5.78515E-10 | 1.44629E-09 |
| ITGA11 | 1.076422118 | 7.35927E-15 | 2.57574E-14 |
| ITGBL1 | 0.681337164 | 5.32884E-07 | 1.03616E-06 |
| ITGB1 | -0.80057725 | 8.85193E-17 | 3.87272E-16 |
| ITGB2 | 0.751348152 | 2.90963E-10 | 7.83361E-10 |
| ITGB3 | -0.572357263 | #N/A | #N/A |
| ITGB4 | -0.471606016 | 0.000218581 | 0.000332623 |
| ITGB5 | 0.274071732 | 0.005246487 | 0.006558109 |
| ITGB6 | 0.102004661 | 0.566077494 | 0.600385221 |
| ITGB7 | 0.758341493 | 2.01663E-08 | 4.41138E-08 |
| ITGB8 | -0.622003402 | 0.000100659 | 0.00016014 |

| **GENE SYMBOL** | **log2FoldChange** | **pvalue** | **padj** |
| --- | --- | --- | --- |
|  |  | **PCPG** |  |
| ITGAD | 2.904276902 | 0.016370177 | 0.077758343 |
| ITGAE | -0.526832159 | 0.22934611 | 0.51265601 |
| ITGAL | 0.524169317 | 0.446548186 | 0.585132105 |
| ITGAM | -1.017352488 | 0.17186839 | 0.466499916 |
| ITGAV | -1.18736366 | 0.069401985 | 0.219772952 |
| ITGAX | 0.892370982 | 0.25648747 | 0.512974941 |
| ITGA1 | -1.593196949 | 0.004666587 | 0.029555051 |
| ITGA2 | -2.659228029 | 0.001239885 | 0.011778909 |
| ITGA2B | 1.660706597 | 0.029507367 | 0.112127995 |
| ITGA3 | 0.597440728 | 0.243971689 | 0.512974941 |
| ITGA4 | 2.356502898 | 0.004359808 | 0.029555051 |
| ITGA5 | -0.680303429 | 0.382182412 | 0.537886357 |
| ITGA6 | 2.76152216 | 4.13088E-05 | 0.001262765 |
| ITGA7 | 0.650203095 | 0.362553546 | 0.537886357 |
| ITGA8 | -0.728933964 | 0.32943813 | 0.537886357 |
| ITGA9 | -2.556716551 | 6.64613E-05 | 0.001262765 |
| ITGA10 | 0.977533501 | 0.222917265 | 0.51265601 |
| ITGA11 | 0.932177952 | 0.294793686 | 0.537886357 |
| ITGBL1 | 0.952319481 | 0.341076301 | 0.537886357 |
| ITGB1 | 0.940214612 | 0.133738019 | 0.390926516 |
| ITGB2 | -0.719828303 | 0.34593319 | 0.537886357 |
| ITGB3 | 3.624146049 | 9.97213E-05 | 0.001263136 |
| ITGB4 | 1.822252641 | 0.019927208 | 0.084137101 |
| ITGB5 | 0.361103635 | 0.586774967 | 0.708151993 |
| ITGB6 | -0.221827792 | 0.820284351 | 0.860796408 |
| ITGB7 | -0.735350472 | 0.373551915 | 0.537886357 |
| ITGB8 | 1.562043499 | 0.011878532 | 0.06448346 |

| **GENE SYMBOL** | **log2FoldChange** | **pvalue** | **padj** |
| --- | --- | --- | --- |
|  |  | **PRAD** |  |
| ITGAD | 1.237405411 | 1.26923E-07 | 2.82052E-07 |
| ITGAE | -0.07932602 | 0.420791322 | 0.480904368 |
| ITGAL | -0.003459446 | 0.985020601 | 0.985020601 |
| ITGAM | -0.381035508 | 0.016271771 | 0.024106327 |
| ITGAV | -0.15392415 | 0.280394802 | 0.329876238 |
| ITGAX | 0.680780453 | 1.38067E-05 | 2.51031E-05 |
| ITGA1 | -1.231298815 | 3.26151E-15 | 1.86372E-14 |
| ITGA2 | -1.487015957 | 9.26194E-19 | 3.70478E-17 |
| ITGA2B | -0.663196322 | 0.000108502 | 0.000188699 |
| ITGA3 | -0.918186544 | 2.05315E-11 | 5.47507E-11 |
| ITGA4 | -0.441074152 | 0.038653586 | 0.053315291 |
| ITGA5 | -1.275274219 | 2.73654E-14 | 9.95105E-14 |
| ITGA6 | -0.62009447 | 3.87932E-06 | 7.75864E-06 |
| ITGA7 | -1.158242991 | 2.05687E-12 | 6.32884E-12 |
| ITGA8 | -1.25134579 | 1.5843E-08 | 3.96076E-08 |
| ITGA9 | -1.408326431 | 2.14125E-17 | 4.2825E-16 |
| ITGA10 | 0.388705216 | 0.009818781 | 0.015105817 |
| ITGA11 | -0.03725613 | 0.841067439 | 0.909262096 |
| ITGBL1 | 1.29532008 | 4.09812E-08 | 9.64263E-08 |
| ITGB1 | -0.989441004 | 4.03418E-12 | 1.15262E-11 |
| ITGB2 | 0.019101244 | 0.910602283 | 0.958528719 |
| ITGB3 | -1.50247208 | 4.66018E-15 | 2.33009E-14 |
| ITGB4 | -1.314437864 | 1.95339E-14 | 7.81355E-14 |
| ITGB5 | -0.129883713 | 0.214510007 | 0.26001213 |
| ITGB6 | -1.778475017 | 4.89745E-17 | 6.52994E-16 |
| ITGB7 | -0.535499815 | 0.006370946 | 0.010577658 |
| ITGB8 | -1.297777165 | 7.01952E-13 | 2.33984E-12 |

| **GENE SYMBOL** | **log2FoldChange** | **pvalue** | **padj** |
| --- | --- | --- | --- |
|  |  | **READ** |  |
| ITGAD | -1.948882977 | 0.000948409 | 0.003698795 |
| ITGAE | -0.342456784 | 0.22826943 | 0.314678952 |
| ITGAL | -1.311291017 | 0.00406958 | 0.010549465 |
| ITGAM | -0.531449692 | 0.269892866 | 0.339542638 |
| ITGAV | -0.316842763 | 0.427405889 | 0.505116051 |
| ITGAX | 0.371098277 | 0.413518367 | 0.50397551 |
| ITGA1 | -1.326677107 | 0.000615029 | 0.002665124 |
| ITGA2 | 0.561626513 | 0.144896916 | 0.209295545 |
| ITGA2B | 0.580604129 | 0.233992041 | 0.314678952 |
| ITGA3 | -0.561888875 | 0.067077011 | 0.109000143 |
| ITGA4 | -1.393484843 | 0.001671944 | 0.005927801 |
| ITGA5 | -1.332807155 | 0.002183584 | 0.007096647 |
| ITGA6 | 0.165669848 | 0.640000647 | 0.713143578 |
| ITGA7 | -2.446266944 | 2.69416E-09 | 3.5024E-08 |
| ITGA8 | -1.692423663 | 8.53093E-05 | 0.000554511 |
| ITGA9 | -0.516804592 | 0.261096656 | 0.339425652 |
| ITGA10 | -0.244167217 | 0.606582132 | 0.695785387 |
| ITGA11 | 1.697345419 | 0.000433632 | 0.002113955 |
| ITGBL1 | 1.897732633 | 0.004327985 | 0.010549465 |
| ITGB1 | -0.790936245 | 0.035064733 | 0.065239818 |
| ITGB2 | -0.927589656 | 0.035129133 | 0.065239818 |
| ITGB3 | -1.737634477 | 1.04329E-05 | 8.13767E-05 |
| ITGB4 | -0.004699977 | 0.987140901 | 0.994456811 |
| ITGB5 | -0.085924649 | 0.787779633 | 0.853427936 |
| ITGB6 | -0.635167586 | 0.10625901 | 0.159388515 |
| ITGB7 | -1.264056542 | 0.003875488 | 0.010549465 |
| ITGB8 | 0.917232288 | 0.044526607 | 0.07893353 |

| **GENE SYMBOL** | **log2FoldChange** | **pvalue** | **padj** |
| --- | --- | --- | --- |
|  |  | **CESC** |  |
| ITGAD | 0.943953311 | #N/A | #N/A |
| ITGAE | 0.924584366 | 0.092853718 | 0.139280576 |
| ITGAL | 1.058374111 | 0.212313027 | 0.272973892 |
| ITGAM | 0.720348331 | 0.360877926 | 0.433053512 |
| ITGAV | 0.210322249 | 0.732278669 | 0.775353885 |
| ITGAX | 1.838062073 | 0.006566494 | 0.018184137 |
| ITGA1 | -1.042354771 | 0.151900068 | 0.210323171 |
| ITGA2 | 2.842488367 | 0.000811238 | 0.002920458 |
| ITGA2B | 1.653495782 | 0.078121313 | 0.13392225 |
| ITGA3 | 1.854532277 | 0.024055581 | 0.059895003 |
| ITGA4 | 0.666938011 | 0.416452145 | 0.468508663 |
| ITGA5 | -1.645036034 | 0.049169131 | 0.091030259 |
| ITGA6 | 0.988223413 | 0.210357364 | 0.272973892 |
| ITGA7 | -3.365183708 | 1.90173E-05 | 0.000171156 |
| ITGA8 | -2.835700526 | 0.002307145 | 0.006921436 |
| ITGA9 | -3.408663982 | 7.42057E-05 | 0.000381629 |
| ITGA10 | -1.862387279 | 0.049463459 | 0.091030259 |
| ITGA11 | -4.550475881 | 6.22064E-06 | 7.46477E-05 |
| ITGBL1 | -0.590615351 | 0.64146353 | 0.699778397 |
| ITGB1 | -1.467047828 | 0.029570881 | 0.06262069 |
| ITGB2 | 1.542271053 | 0.050572366 | 0.091030259 |
| ITGB3 | -2.588586204 | 0.026698072 | 0.060070663 |
| ITGB4 | 2.33081203 | 0.000894157 | 0.002926331 |
| ITGB5 | -0.576592359 | 0.385953437 | 0.448203991 |
| ITGB6 | 5.906419377 | 2.55595E-11 | 7.2112E-10 |
| ITGB7 | 2.575213719 | 0.000751759 | 0.002920458 |
| ITGB8 | 1.204288243 | 0.082645162 | 0.135237537 |

| **GENE SYMBOL** | **log2FoldChange** | **pvalue** | **padj** |
| --- | --- | --- | --- |
|  |  | **STAD** |  |
| ITGAD | 1.651731668 | 7.54595E-08 | 2.35927E-07 |
| ITGAE | 0.788576473 | 9.3965E-07 | 2.17294E-06 |
| ITGAL | 0.804279229 | 0.002101752 | 0.002990954 |
| ITGAM | 1.224174606 | 5.89677E-07 | 1.45454E-06 |
| ITGAV | 0.683393699 | 0.000222354 | 0.0003577 |
| ITGAX | 2.362860763 | 7.28139E-24 | 2.69411E-22 |
| ITGA1 | -0.708020096 | 0.002744523 | 0.003761013 |
| ITGA2 | 1.825468775 | 2.87797E-16 | 3.5495E-15 |
| ITGA2B | 0.081752398 | 0.749509686 | 0.749509686 |
| ITGA3 | 0.864279161 | 4.22582E-05 | 8.22923E-05 |
| ITGA4 | 1.013298431 | 2.24113E-05 | 4.60678E-05 |
| ITGA5 | 0.486754338 | 0.042088584 | 0.053699228 |
| ITGA6 | 1.442197699 | 2.85112E-12 | 2.63729E-11 |
| ITGA7 | -1.126211898 | 0.000139375 | 0.000245565 |
| ITGA8 | -1.849555394 | 4.01927E-09 | 2.47855E-08 |
| ITGA9 | -0.969113804 | 0.000253186 | 0.000390329 |
| ITGA10 | 0.464800845 | #N/A | #N/A |
| ITGA11 | 2.226117311 | 2.16501E-18 | 4.00526E-17 |
| ITGBL1 | 1.58385885 | 3.30281E-06 | 7.18848E-06 |
| ITGB1 | 0.637139468 | 0.000444092 | 0.000657256 |
| ITGB2 | 1.351251397 | 1.4863E-08 | 5.49931E-08 |
| ITGB3 | 0.27359814 | 0.254566057 | 0.285422549 |
| ITGB4 | 1.405474363 | 1.14215E-09 | 8.45191E-09 |
| ITGB5 | 0.676386279 | 0.000112021 | 0.000207239 |
| ITGB6 | 1.597468518 | 8.28932E-08 | 2.35927E-07 |
| ITGB7 | -0.341210033 | 0.162396171 | 0.200244858 |
| ITGB8 | 1.332233371 | 1.22823E-08 | 5.04939E-08 |
